# Construction of porphyrinic manganese-organic frameworks based on structural regulation for electrochemical determination of nitrobenzene in water and vegetable samples

**DOI:** 10.3389/fchem.2024.1380551

**Published:** 2024-03-20

**Authors:** Li Wang, Mengjie Zhang, Yuanyuan Li, Xiumei Chen, Hao Qin, Jin Yang, Suhua Fan, Hai Wu

**Affiliations:** Anhui Province Key Laboratory for Degradation and Monitoring of Pollution of the Environment, Anhui Province Key Laboratory of Environmental Hormone and Reproduction, Fuyang Normal University, Fuyang, China

**Keywords:** metal-organic frameworks, nitrobenzene, electrochemical sensor, organic pollutants, structural regulation

## Abstract

Nitrobenzene (NB) is one of the major organic pollutants that has seriously endangered human health and the environment even in trace amounts. Therefore, it is of great significance to detect trace NB efficiently and sensitively. Herein, a porphyrinic metal-organic framework (MOF) of Mn-PCN-222 (PCN, porous coordination network) was first synthesized by the coordination between Zr_6_ cluster and tetrakis (4-carboxyphenyl)-porphyrin-Mn (Ⅲ) (MnTCPPCl) ligand. To regulate its structure and the electrochemical properties, a phenyl group was inserted in each branched chain of TCPP to form the TCBPP organic ligand. Then, we used Zr_6_ clusters and manganese metalloporphyrin (MnTCBPPCl) to synthesize a new porphyrin-based MOF (Mn-CPM-99, CPM, crystalline porous material). Due to the extended chains of TCPP, the rod-shaped structure of Mn-PCN-222 was switched to concave quadrangular bipyramid of Mn-CPM-99. Mn-CPM-99 exhibited higher porosity, larger specific surface area, better electrochemical performances than those of Mn-PCN-222. By using modular assembly technique, Mn-CPM-99 film was sequentially assembled on the surface of indium-tin-oxide (ITO) to prepare an electrochemical sensor (Mn-CPM-99/ITO). The proposed sensor showed excellent electrochemical reduction of NB and displayed three linear response ranges in the wide concentration ranges. The obtained low limit of detection (LOD, 1.3 nM), high sensitivity and selectivity, and good reproducibility of the sensor for NB detection fully illustrate that Mn-CPM-99 is an excellent candidate for electrochemical sensor interface material. Moreover, the sensor was successfully applied to the detection of NB in lake water and vegetable samples showing satisfactory recovery of 98.9%–101.8%.

## 1 Introduction

Nitrobenzene (NB) and its compounds play crucial roles in the synthesis of dyes, pesticides, and explosives, moreover, they serve as solvents during petroleum refining processes (Li et al., 2022). NB contamination in the environment is mainly from the discharge of industrial waste by chemical plants and dyestuff factories (Wang et al., 2023). Due to its moderate water solubility and higher density relative to water, NB compounds tend to sink to the bottom of water and persist for a prolonged period, resulting in severe and persistent pollution of natural water resources (Lu et al., 2023; Wang et al., 2024). When NB enter into the human body, it can lead to a serious problem of methemoglobinemia and liver cancer and remain carcinogenic activities even at trace level (Fang et al., 2021; Li et al., 2021). Moreover, the presence of a nitro group in NB compounds contributes to their poor biodegradability in electron-deficient environments. The allowable level of NB in all types of water is regulated no more than 2 mg⋅L^−1^ (16.25 μM) by the World Health Organization (WHO) and American Public Health Association (APHA) (Li et al., 2023). It is therefore imperative to enhance environmental monitoring and research on nitrobenzene compounds, implement stricter emission controls, mitigate environmental pollution, and safeguard human health (Seal et al., 2022; Chellappa et al., 2024).

Various analytical methods including gas chromatography-mass spectrometry (GC-MS) (Zhang et al., 2014), high performance liquid chromatography (HPLC) (Wang and Chen, 2002), spectrophotometry (Rafique et al., 2023; Rahman and Ahmad, 2024) and electrochemical techniques have been used for the detection of NB (Koohfar et al., 2023; Wang et al., 2023; Papavasileiou et al., 2024). Among them, electrochemical techniques have received widespread attention due to their simple operation, high sensitivity, and low cost for detecting nitrobenzene. However, the key of electrochemical sensors is to prepare the interface materials with excellent selectivity and good catalytic performance. Metal-organic frameworks (MOFs) are hybrid materials composed of both organic linkers and inorganic metal nodes, and have been extensively used to construct electrochemical sensors (Kajal et al., 2022). MOFs possess special active sites and many advantages due to their controllable structure, high specific surface area, good stability, and tailored functionalities (Zhang et al., 2023; Junior et al., 2024). The composition of organic and inorganic components can be adjusted to control the structure and performances of MOFs, enhancing their sensing capabilities, e. g., by incorporating specific functional groups for target recognition to realize selective detection of target analytes (Liu et al., 2020; Chang et al., 2022). Therefore, several electrochemical sensors based on MOFs have been developed including Cu-MOF (Xin et al., 2023), PCN-222(Fe) (Li et al., 2023), Fe-MOF (Dhayanithi et al., 2023) and showed outstanding sensitivity for the detection of analytes. The adjustable structure and chemical properties make MOFs and their derived materials promising candidates for the development of advanced electrochemical sensors.

Porphyrin-based MOFs can selectively response to NB compounds due to the inherent porphyrin recognition sites in MOFs, exhibiting rapid and selective detection of NB compounds, even in the presence of other interfering compounds (Yang et al., 2015; Zhang et al., 2021). A Zr (IV)-porphyrin MOF PCN-224 (PCN, porous coordination network) was used as fluorescent sensor for rapid detection of 2,4,6-trinitrotoluene (TNT) in aqueous solution (Yang et al., 2015). Fischer et al. proposed an electrochemical sensor using a porphyrin-based MOF (Mn-PCN-222) modified on a conductive indium-tin-oxide (ITO) surface for a variety of electrochemical applications, including nitroaromatics, phenolic and quinone-hydroquinone toxins, heavy metal ions, and biological species (Zhou et al., 2021). The excellent features were attributed to their well-defined porous structures, good electron transfer, and selective reaction between tetra(4-carboxyphenyl)porphyrin (TCPP) and nitroaromatics through hydrogen bonding and π−π stacking interaction.

**SCHEME 1:**
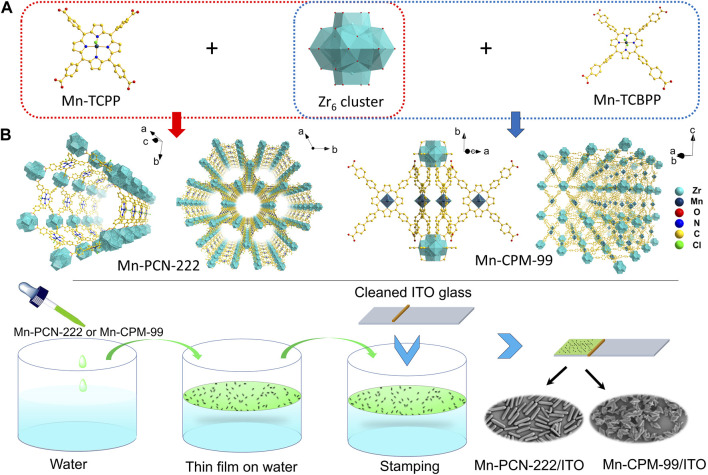
**(A)** Synthesis routs and structures of Mn-PCN-222 and Mn-CPM-99. **(B)** Schematic diagram for the processes of modular assembly of Mn-PCN-222 and Mn-CPM-99 on the ITO electrodes.

In this research, to utilize the advantages of porphyrin-based MOFs, we employed Zr_6_ clusters as nodes and manganese metalloporphyrin as ligand to prepare a new porphyrin-based MOF (Mn-CPM-99, CPM, crystalline porous material). As shown in Scheme 1A, Zr_6_ cluster and Mn-TCPP were first coordinated to construct Mn-PCN-222, containing one of the 1D hexagonal mesoporous channels (Feng et al., 2012; Zhou et al., 2021). To regulate its structure, a phenyl group was inserted in each branched chain of Mn-TCPP to form the Mn-TCBPP ligand, and then, Mn-CPM-99 was prepared. Due to the extended chains of TCPP, the rod-shaped structure of Mn-PCN-222 was switched to quadrangular bipyramid of Mn-CPM-99. Their chemical structures, morphologies, electrochemical behavior, and electrocatalytic abilities were compared in this study. The results showed that the Mn-CPM-99 based electrochemical sensor exhibited high sensitivity and good performances for detection of NB in water and vegetable.

## 2 Experiments

### 2.1 Chemicals and reagents

All chemicals and reagents including N,N-Dimethylformamide (DMF), N,N-Diethylformamide (DEF), tetrahydrofuran (THF), were purchased from Energy Chemical (Shanghai, China) and Aladdin Reagent Shanghai Co. Ltd. (China), and directly utilized without further purification. The synthesis processes and structural characterizations for the Manganese porphyrin Lingands of MnTCPPCl and MnTCBPPCl Ligands were provided in Supplementary Scheme S1; Supplementary Figures S1–S3). Purification of synthesized products was performed on silica gel (300–400 mesh) and self-prepared PTLC (GF254 silica gel).

### 2.2 Synthesis of Mn-PCN-222 and Mn-CPM-99 MOFs

Mn-PCN-222 was synthesized by a simple one-step solvothermal method (Zhou et al., 2021). ZrOCl_2_-8H_2_O (26 mg), MnTCPPCl (12 mg), and benzoic acid (225 mg) were dissolved in 2 mL of DMF, and ultrasonically dispersed for 10 min. The reaction was then heated with stirring at 80°C and kept for 24 h. When the reaction was cooled to room temperature, the resulting Mn-PCN-222 were rinsed and centrifuged by DMF and ethanol, respectively, and finally dried under vacuum. In the similar way, Mn-CPM-99 were synthesized in DEF solvent at 120°C using 12 mg of MnTCBPPCl ligand.

### 2.3 Preparation of MOFs film modified ITO electrodes

Mn-PCN-222 and Mn-CPM-99 films was modified on the surface of conductive indium-tin-oxide (ITO) via the modular assembly method. As shown in Scheme 1B, before the modification, the ITO glass was cleaned in ultrasonic baths of acetone, ethanol, and water for 20 min, respectively. Then cleaned ITO was immersed in a solution of (1:1 v/v) ethanol/NaOH (1 M) and was activated for 15 min. Finally, it was rinsed with pure water and dried by N_2_. The synthesized Mn-PCN-222 or Mn-CPM-99 were homogeneously dispersed in ethanol with ultrasonication to obtain a colloidal suspension of 1.0 mg⋅mL^-1^, which was dropped onto the water surface in a beaker and then spread out to form a thin film. The film was then transferred onto the cleaned ITO glass by stamping (1 cm × 1 cm). The assembled film was immersed in ultrapure water to remove the un-deposited Mn-PCN-222 or Mn-CPM-99 and then dried by using N_2_, Finally, the Mn-PCN-222/ITO and Mn-CPM-99/ITO modified electrodes were obtained.

### 2.4 Apparatus and measurements

Cyclic voltammetry (CV), chronoamperometric technique (i–t), and electrochemical impedance spectroscopy (EIS) were performed on a CHI660E electrochemical workstation (Chenhua, Shanghai, China). A three-electrode system was used and consisted of a modified ITO electrode as the working electrode (1 cm × 1 cm), a platinum wire served as the auxiliary electrode and an Ag/AgCl electrode (3 M KCl) used as the reference electrode, respectively.

The morphology, composition, elements, and chemical structure of Mn-PCN-222 and Mn-CPM-99 were characterized by Scanning electron microscope (SEM, Sigma 500, Carl Zeiss, Germany), Transmission electron microscope (TEM, Tecnai G2 F30, FEI, USA), powder X-ray diffraction (XRD, SmartLab SE, Rigaku, Japan), X-ray photoelectron spectroscopy (XPS, K-Alpha, Thermo Scientific, USA), Attenuated total reflectance Infrared spectroscopy (ATR-IR, Nicolet iS50, Thermo Fisher, USA), and UV-Vis spectroscopy (UV-2700i spectrometer, Shimadzu, Japan). ^1^H NMR spectra for the characterization of the Mn-PCN-222 and Mn-CPM-99 structures in Supplementary Material were measured on a Bruker Ascend 400 MHz instrument (NMR, Ascend 400, BRUKER, Switzerland).

## 3 Results and discussion

### 3.1 Characterization of Mn-PCN-222 and Mn-CPM-99

As described in Supplementary Scheme S1, the intermediates of TPPCOOMe and H_2_TEtCBPP were synthesized and characterized by ^1^H NMR spectra, and they were further coordinated with manganese metal ions to obtain MnTCPPCl and MnTCBPPCl ligands, respectively (Supplementary Figures S1–S3). MnTCPPCl and MnTCBPPCl were then hydrolyzed to produce side-chain ester groups, which were then reacted with Zr_6_ clusters by solvothermal synthesis method to get the Mn-PCN-222 and Mn-CPM-99 MOFs, respectively. The sizes and morphologies of MnTCPPCl and MnTCBPPCl were observed by SEM and TEM measurements, respectively.

**FIGURE 1:**
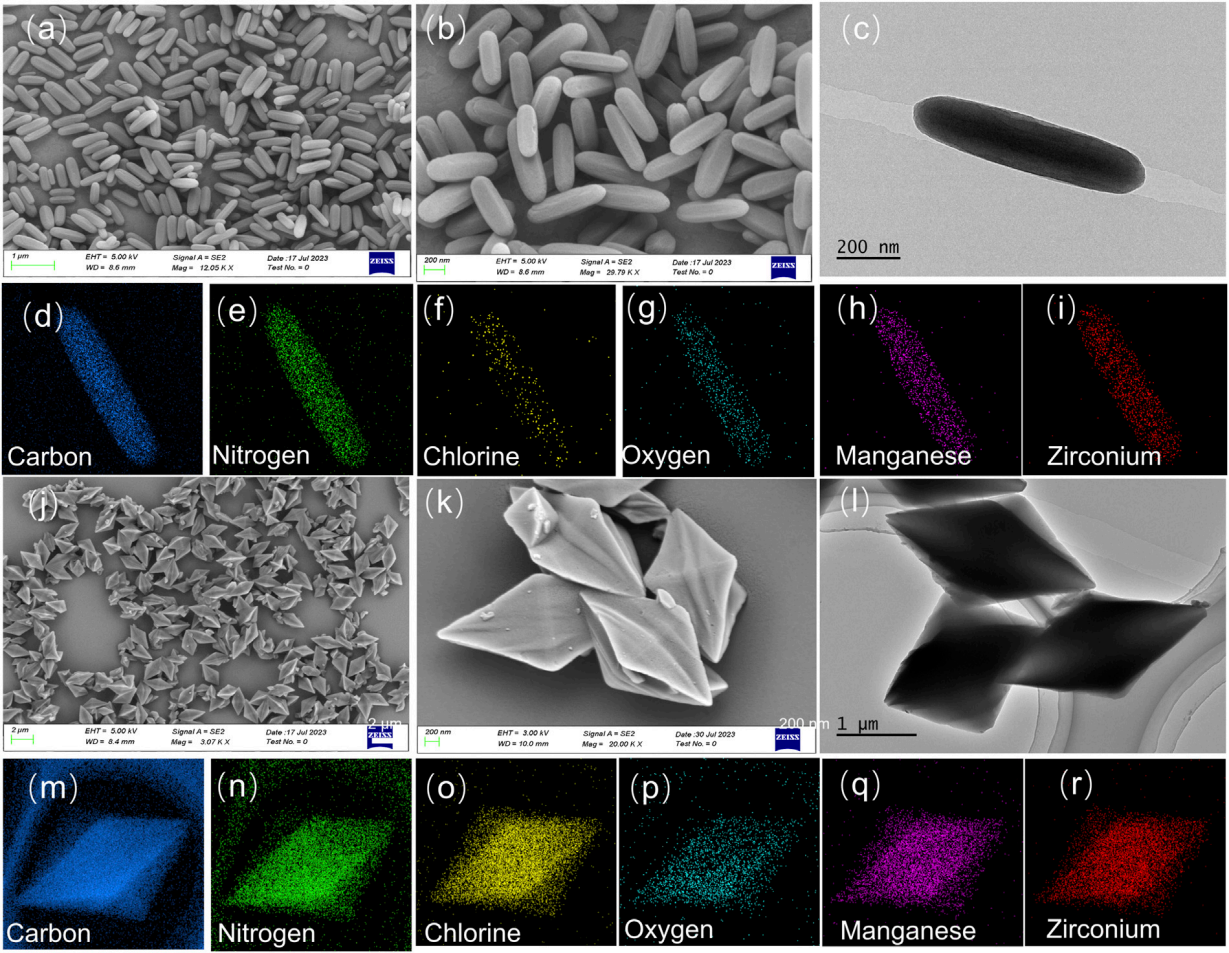
SEM images of Mn-PCN-222 **(A,B)** and Mn-CPM-99 **(J,K)**. TEM images of Mn-PCN-222 **(C)** and Mn-CPM-99 **(L)**, respectively. Element mapping images of Mn-PCN-222 **(D–I)** and Mn-CPM-99 **(M–R)**, respectively.

As shown in Figures 1A–C, Mn-PCN-222 presents regular rod shape with an average diameter of 165 nm. However, Mn-CPM-99 exhibits the structure of concave quadrangular bipyramid with uniform distribution (about 700–850 nm long, Figures 1J–L). The Energy-dispersive X-ray spectroscope (EDS) elemental mapping of Mn-PCN-222 and Mn-CPM-99 MOFs were performed to reveal the element distribution. The EDS spectra presented in Supplementary Figure S4 confirm the presence of C, N, Cl, O, Mn, and Zr elements in the samples. We can find that all the measured elements (C, N, Cl, O, Mn, Zr) in the elemental maps are uniformly distributed in the samples (Figures 1D–I; Figures 1M–R). The clean and homogeneous surfaces of Mn-PCN-222 and Mn-CPM-99 MOFs indicate that the synthesis was successful and could obtain relatively pure compounds.

**FIGURE 2:**
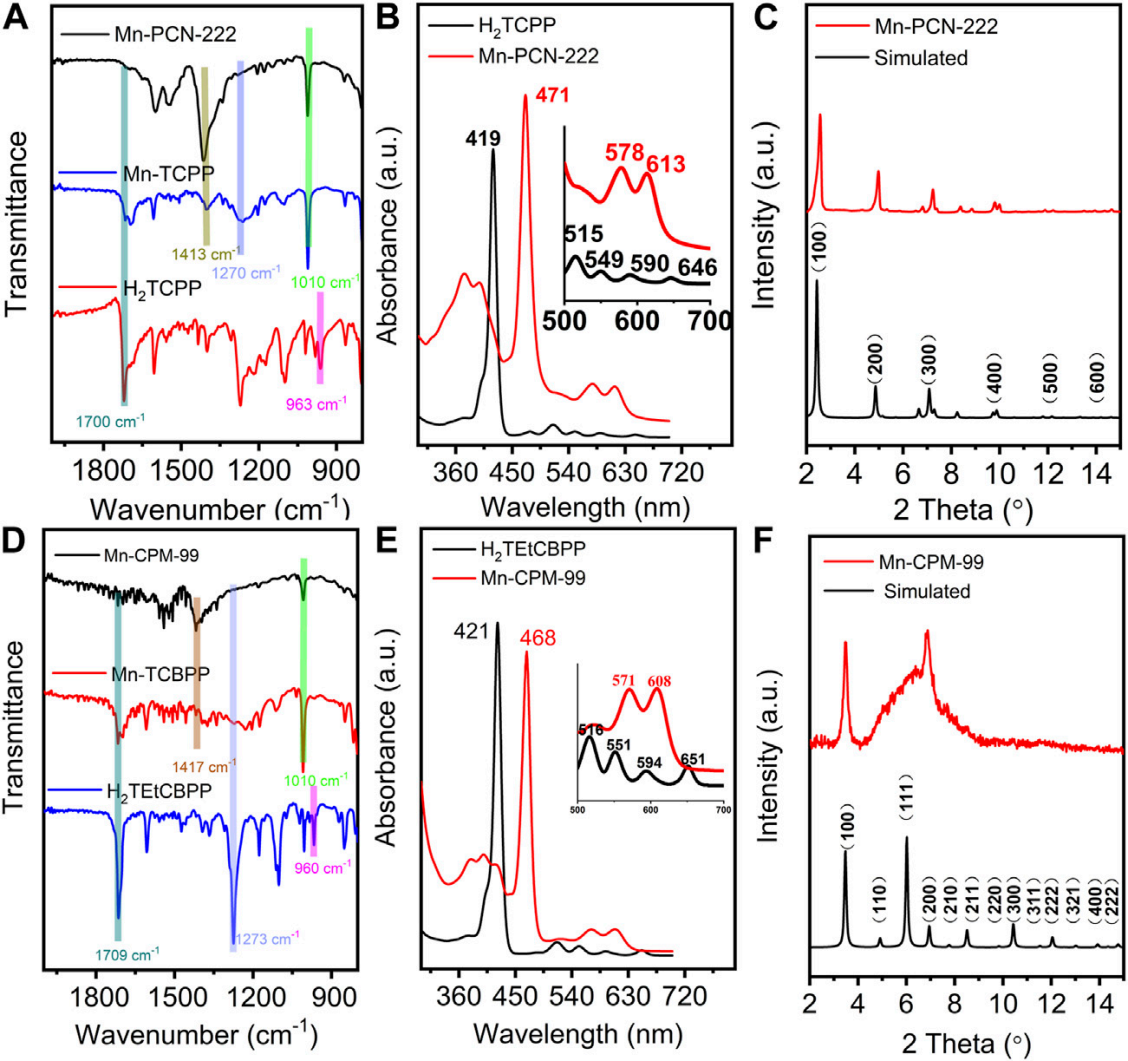
**(A,D)** ATR-IR spectra of free H_2_TCPP ligand, MnTCPPCl ligand, Mn-PCN-222, free H_2_TEtCBPP ligand, MnTCBPPCl ligand, and Mn-CPM-99. **(B,E)** UV-Vis spectra of free H_2_TCPP ligand and Mn-PCN-222 and free H_2_TEtCBPP ligand and Mn-CPM-99 (Inset presents a zoomed view of the enlarged Q-band region for clarity). **(C,F)** PXRD patterns of the simulated (red curves) and prepared (blue curves) Mn-PCN-222 and Mn-CPM-99, respectively.

The compositions of Mn-PCN-222 and Mn-CPM-99 were analyzed by attenuated total reflection infrared spectroscopy (ATR-IR) and UV-Vis spectroscopy. As shown in Figure 2A, compared with the free H_2_TCPP ligand, MnTCPPCl ligand exhibits a new peak at 1010 cm^-1^ assigned to Mn-N bonds with the disappearance of the N-H stretching vibration peak at 963 cm^-1^, indicating the coordination of Mn to porphyrin ring in MnTCPPCl (Yu et al., 2020; Xu et al., 2021; Zhou et al., 2021). The signature of Mn-N bonds is also observed in the Mn-PCN-222 film. In contrast to MnTCPPCl, Mn-PCN-222 does not show the characteristic peaks of C=O bonds (1700 cm^-1^) and C−O bonds (1270 cm^-1^) whereas exhibits strong peak of −COO symmetric stretch bonds (1413 cm^-1^), suggesting the carboxyl group coordinating to the Zr_6_ centers in MnTCPPCl (Figure 2D). The results are agreement with those in the literature and which suggested the Mn-PCN-222 material was prepared successfully (Zhou et al., 2021). Similarly, the ATR-IR spectra of Mn-CPM-99 does not show the characteristic peaks near 1709 cm^-1^ (C=O bond) and 1273 cm^-1^ (C-O bond) of MnTCBPPCl and H_2_TEtCBPP, while the emergence of a strong peak at 1417 cm^-1^ (COO symmetric stretching bond) reflects the carboxyl group coordinated to the Zr_6_ cluster in MnTCBPPCl (Zhou et al., 2021; Zhu et al., 2021; Kaur et al., 2023). In addition, the metallation of the MnTCBPPCl ligand can be proved by the disappearance of the N-H stretching vibration at 960 cm^-1^ and the appearance of a new peak for the Mn-N bond at 1010 cm^-1^. The UV-Vis spectra of both H_2_TCPP and H_2_TEtCBPP ligands present a strong Soret band and four Q-bands (Figures 2B, E). Furthermore, the Soret bands of Mn-PCN-222 and MnTCBPPCl films are noticed to undergo red shifts about 50 nm, and only two Q bands can be observed, confirming the metalation of porphyrin rings by Mn(III) in the Mn-PCN-222 films. Moreover, there is no characteristic peak of the free TCPP or other new metallized TCPP, indicating the absence of Mn leaching and substitution by other metal ions during the synthesis of Mn-PCN-222 films.

Powder X-ray diffraction (PXRD) patterns of Mn-PCN-222 and Mn-CPM-99 were recorded in Figures 2C, F. The results show that the intense diffraction peaks of Mn-PCN-222 are in good agreement with the simulated XRD profile, which indicate that the three-dimensional Mn-PCN-222 has been successfully prepared. Figure 2F displays the XRD patterns of Mn-CPM-99 which the plane of (100) is located at 2θ of 3.5°. Meanwhile, the broad peak around 2θ of 7° is attributed to the X-ray diffraction peak broadening by amorphization effect. These results indicate the existence of nanocrystals with tiny size in the structure of Mn-CPM-99. More evidences in Supplementary Figure S5 exhibit that Mn-CPM-99 are combined by the nanocrystals sizing of ca. 5.9 nm and organic ligands with disordered structures. Further, the calculated interplanar spacing of the nanocrystals is about 1.28 nm which is corresponding to the plane of (200) locates at 6.94° (Kollias et al., 2022; Xu et al., 2022). Then, the highly dispersed diffraction ring in Supplementary Figure S6 of the selected electron diffraction pattern confirms Mn-CPM-99 possess typical amorphous structure. The abovementioned results are identical to the conclusion of the XRD pattern for Mn-CPM-99.

**FIGURE 3:**
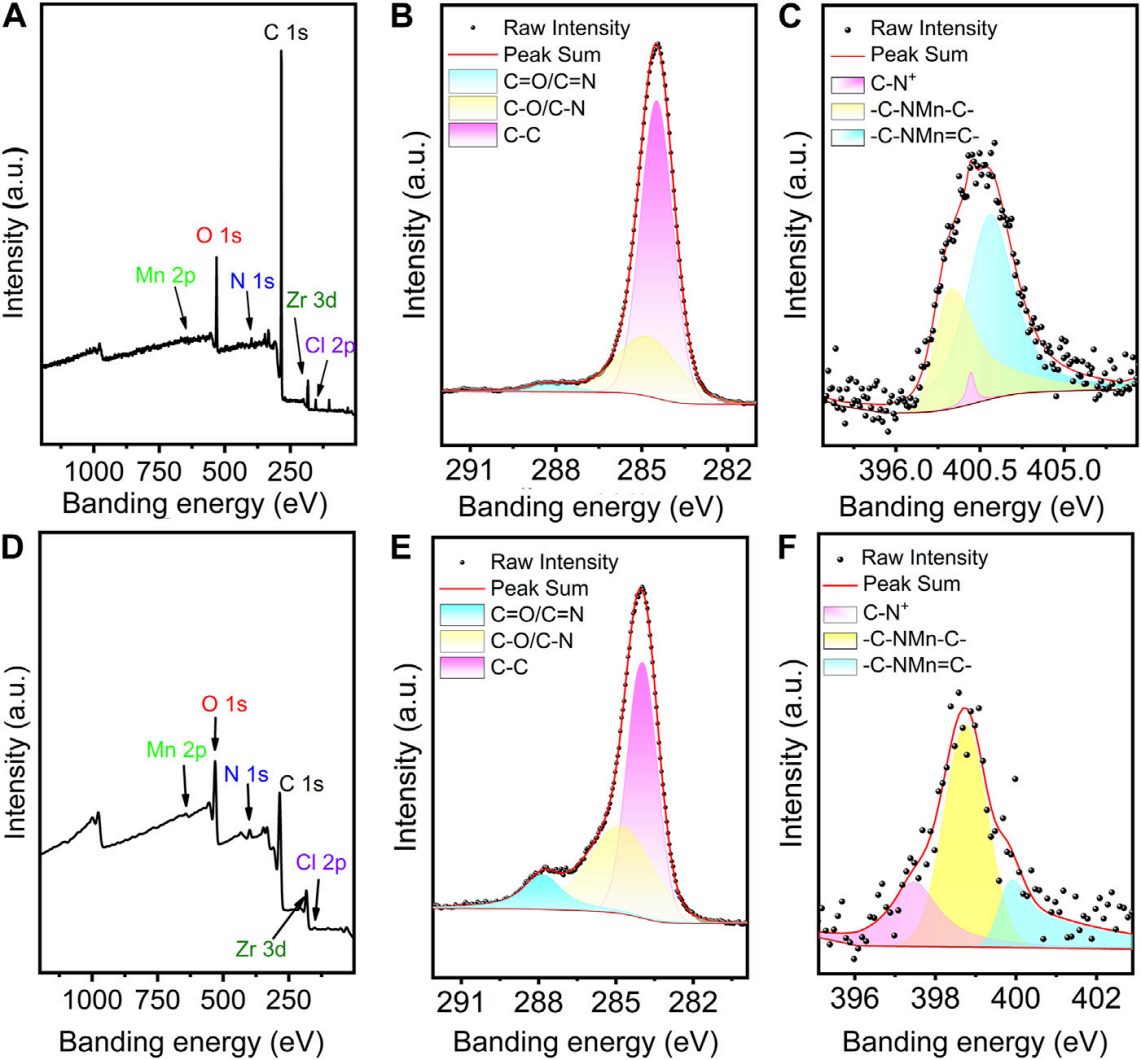
Full XPS profiles of Mn-PCN-222 **(A)** and Mn-CPM-99 **(D)**. The high-resolution XPS spectra of C 1s **(B,E)** and N 1s **(C,F)** in Mn-PCN-222 and Mn-PCN-222, respectively.

The elemental valence states of Mn-PCN-222 and Mn-CPM-99 materials were investigated using XPS analysis, respectively. The full XPS survey spectra are shown in Figures 3A, D and demonstrate the presence of the C, N, Cl, O, Mn, and Zr elements in the Mn-PCN-222 and Mn-CPM-99 materials, which are in good agreement with the above-mentioned results of the EDX energy spectra. In Figure 3B, the three peaks at 288.07 eV, 284.80 eV, and 284.48 eV in the C 1s spectra are attributed to the C-O/C-N, C-C, C=O/C=N valence bonding structure of the TCPP framework, respectively. Correspondingly, the three resolved peaks from the C 1s spectra at 287.81 eV, 284.80 eV and 283.96 eV are attributed to the C-O/C-N, C-C, C=O/C=N valence bond structures of the TCBPP framework, respectively (Figure 3E). Figures 3C, F show the peaks at 399.98, 398.98 and 401.18 eV in Mn-PCN-222 and 397.47, 398.75, and 399.89 eV in Mn-CPM-99 for the N 1s, corresponding to C-N, C-N-Mn-C, and C-N-Mn=C, respectively. Both of the Mn 2P XPS spectra of the Mn-PCN-222 (Supplementary Figure S7A) and Mn-CPM-99 (Supplementary Figure S7B) show four peaks for two oxidation states of Mn(III) and Mn(II) in 2P_1/2_ and 2P_3/2_ bimodal states, respectively. The amplified spectrum of O 1s (Supplementary Figure S7C, D) can be deconvoluted into three peaks (531.06, 532.3 and 529.65 eV in Mn-PCN-222; 532.5, 531.9 and 530.37 eV in Mn-CPM-99), which are attributed to the C=O, Zr-O, and C-O valence bond structures, respectively. These results confirm the successful preparation of Mn-PCN-222 and Mn-CPM-99.

### 3.2 Electrochemical properties of the different modified electrodes

**FIGURE 4:**
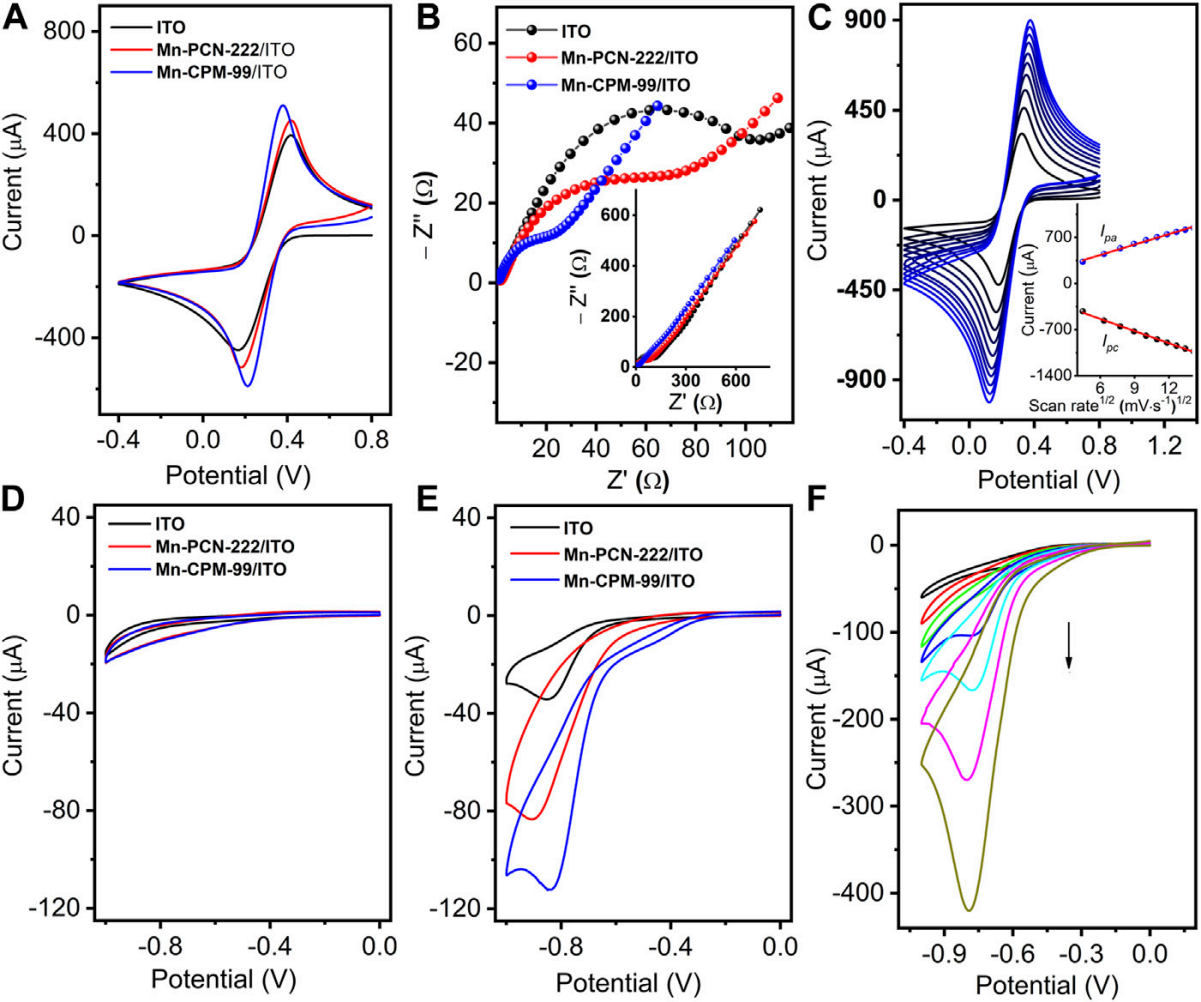
**(A)** CV and **(B)** EIS of ITO, Mn-PCN-222/ITO, and Mn-CPM-99/ITO in 5 mM [Fe(CN)_6_]^3−/4−^ solution with 0.1 M KCl (scan rate: 50 mV⋅s^−1^). **(C)** CVs of Mn-CPM-99/ITO at different scan rates from 20 to 200 mV⋅s^−1^ (Inset: The linear relationship between redox peak currents (*I*_pa_ and *I*_pc_) and the square root of the scan rates). **(D,E)** CVs of different modified electrodes in 0.4 M NaCl (pH 7.0) without and with 40 μM NB, respectively. **(F)** CV response of the Mn-CPM-99/ITO sensor in 0.4 M NaCl toward various concentration of NB (0.06, 0.3, 3, 24, 60, 150, and 300 μM).

Mn-PCN-222 and Mn-CPM-99 were rapidly integrated on ITO glass by modular assembly method to obtain the Mn-PCN-222/ITO and Mn-CPM-99/ITO modified electrodes, which were characterized by CV technology and electrochemical impedance method. The CV curves of the ITO, Mn-PCN-222/ITO, and Mn-CPM-99/ITO electrodes were recorded in 5 mM [Fe(CN)_6_]^3−/4−^ solution with 0.1 M KCl at a scan rate of 50 mV⋅s^−1^ (Figure 4A). Compared to the ITO and Mn-PCN-222/ITO electrodes, the Mn-CPM-99/ITO electrode shows the best reversible redox peaks and largest peak currents. All their corresponding Nyquist plots in 5 mM [Fe(CN)_6_]^3−/4−^ solution Figure 4B show very small resistivity (*R*_ct_). But the *R*_ct_ value of the Mn-CPM-99/ITO electrode (34 Ω) is significantly lower than those of Mn-PCN-222/ITO (86 Ω) and ITO (124 Ω). The results can be attributed to the strong synergistic effect between the organic ligand and the zirconium clusters after ring extension, resulting in more active sites, lower charge transfer resistance, and a larger contact area between the electrode and electrolyte. The CV curves of the Mn-CPM-99/ITO, ITO, and Mn-PCN-222/ITO electrodes at different scan rates (20–200 mV⋅s^−1^) are shown in Figure 4C and Supplementary Figure S8. There are good linear relationships between the redox peak currents and the square root of the scan rates. The good linearity indicates that the redox process of the [Fe(CN)_6_]^3−/4−^ probe is a diffusion-controlled process at the modified electrodes. According to the Randles-Sevcik equation (Supplementary Figure S8), the electrochemically active surface areas of ITO, Mn-PCN-222/ITO and Mn-CPM-99/ITO were calculated to be 0.353, 0.497, and 0.558 cm^2^, respectively, and therefore, the significantly larger active surface area of Mn-CPM-99/ITO provides obvious advantages for NB detection.

To investigate the catalytic activities, the CV responses of the modified electrodes were recorded in 0.4 M NaCl. As shown in Figure 4D, in the absence of NB, three different electrodes do not show obvious reduction peaks, but show different capacitive characteristics from ITO, Mn-PCN-222/ITO, and Mn-CPM-99/ITO modified electrodes due to their porosity and specific surface area. In the presence of NB, Mn-CPM-99/ITO shows sharp and well-defined reduction peak with the highest peak currents and more positive reduction potentials compared to other modified and unmodified electrodes (Figure 4E). Based on the special structure of the manganese metal porphyrin-based MOFs, the higher electrocatalytic activity for NB can be ascribed to reasons as follows: Firstly, the highly conjugated porphyrin ring (π−π stacking interaction with the NB molecules) and the large specific surface of Mn-PCN-222 and Mn-CPM-99 as the porous substrates enable the sensor interface to effectively trap NB molecules; Secondly, the inherent redox activity of the organic ligands TCPP and TCBPP, and the reduction of the electron intermediate of Mn(III) to Mn(II) state in the center of Mn-PCN-222 and Mn-CPM-99 structure contribute to the acceleration of electron transport, which were confirmed by the electrocatalysis of NB on TCPP/ITO, TCBPP/ITO, MnTCPP/ITO, and MnTCBPP/ITO electrodes (Supplementary Figure S9). In addition, the strong electron-withdrawing property of NB itself leads to the formation of an electron donor-acceptor (EDA) system between the porphyrin centers, which facilitates the transfer of electrons to the nitrobenzene, causing its protonation and ultimately leading to the reduction of NB (Zhou et al., 2021).

**FIGURE 5:**
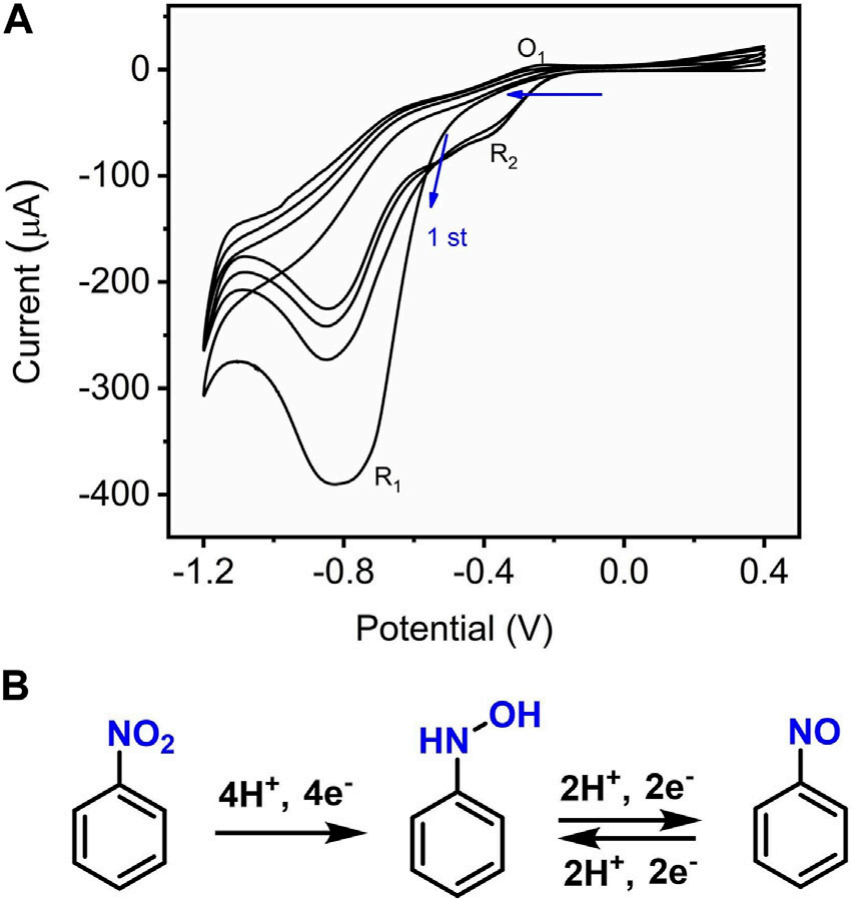
**(A)** CVs of Mn-CPM-99/ITO in 0.4 M NaCl (pH 7.0) containing 0.3 mM NB for four potential cycle scans; **(B)** Electrochemical redox processes of NB on the interface of Mn-CPM-99/ITO.

Figure 4F shows a typical CV response of the Mn-CPM-99/ITO sensor upon continuous addition of NB in 0.4 M NaCl solution. The dramatic increase in the reduction peak currents of NB suggested that the presented sensor showed typical electrocatalytic reduction of NB. In order to study the electrochemical process, CV of 0.5 mM NB was continuously scanned with a wider potential range between 0.4 V and −1.2 V (Figure 5A). During the first sweep, CV of NB at Mn-CPM-99/ITO presents only an irreversible cathodic peak (R_1_) at −0.8 V, which is related to the 4H^+^/4e^−^ reduction of the nitro group (−NO_2_) in NB to phenylhydroxylamine. Starting from the second cycle scan, the reduction peak at −0.83 V gradually decreases, and a pair of new redox peaks (O_1_/R_2_) gradually appear and increase between −0.2 V and −0.4 V, which corresponds to is the 2H^+^/2e^−^ redox process between phenylhydroxylamine and nitrosobenzene (Li et al., 2023; Yuan et al., 2023). The results suggested that phenylhydroxylamine was gradually produced by electrochemical reduction of NB, and then was oxidized to nitrosobenzene, appearing a new reduction peak (R_2_). The overall redox mechanism of NB is summarized in Figure 5B.

### 3.3 Effect of the electrolyte solutions and pH

**FIGURE 6:**
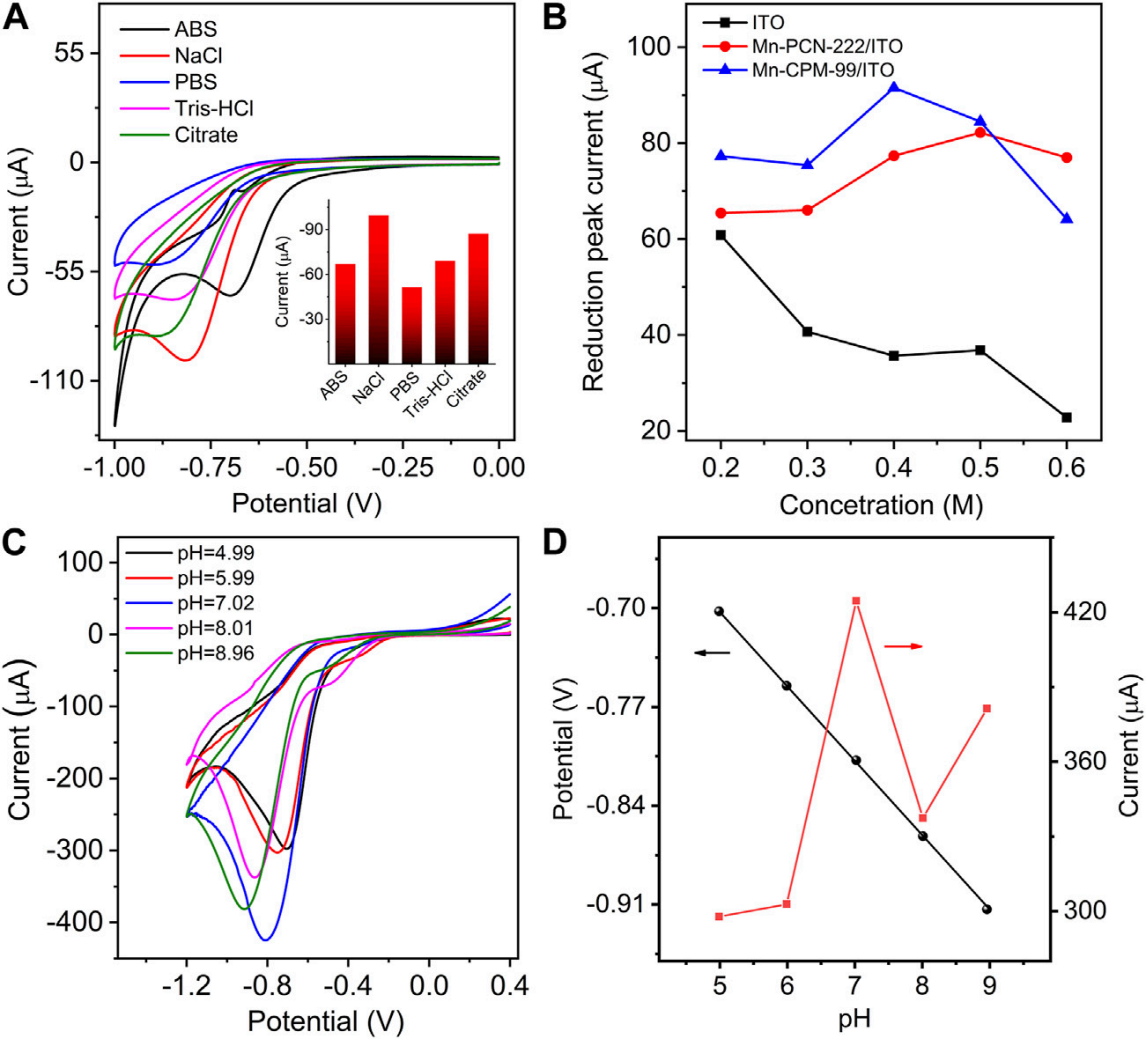
**(A)** CVs of Mn-CPM-99/ITO in different electrolyte solutions in the presence of 40.0 μM NB. (ABS: acetic acid buffer solution, PBS: phosphoric acid buffer solution, Tris-HCl: Tris (hydroxymethyl) aminomethane hydrochloride, Citate: Sodium citrate-citric acid buffer solutions; Concentration of every solution: 0.4 M). Inset: The reduction peak current in different electrolyte solutions. **(B)** The reduction peak currents of NB at different electrodes in NaCl solution with various concentration from 0.20 to 0.60 M **(C)** CVs of Mn-CPM-99/ITO toward 0.30 mM NB of in 0.40 mM NaCl with different pH. **(D)** Change in the reduction peak potential and current at different pH obtained from **(C)**.

To achieve the best electrochemical response, the effects of different electrolyte solutions, electrolyte concentration, and pH of the solution on the electrocatalysis of the sensor toward NB were investigated (Sang et al., 2014; Stergiou et al., 2022). In different electrolyte and buffer solutions, the best response of the Mn-CPM-99/ITO electrode toward NB was obtained in NaCl solution (Figure 6A and inset). Figure 6B and Supplementary Figure S10 show the electrochemical reduction of 40 μM NB in NaCl solution with various concentrations from 0.2 M to 0.6 M. Obviously, the maximum current of NB could be obtained in 0.4 M NaCl solution. Therefore, 0.4 M NaCl solution was chose as an electrolyte concentration in the following study.

Figure 6C shows the dependence of the sensor on pH. The reduction peak potential shifted negatively with increasing pH. The maximum electrocatalytic current was observed at pH seven and a good linear relationship between peak potential and pH was obtained: Ep (V) = −0.0529 pH−0.438 (Figure 6D). The slope of −0.0529 V/pH is very close to the theoretical value of 0.059 V/pH. The result indicates that an equal number of electrons and protons is involved in the NB electrochemical reduction process (Yuan et al., 2023), which is consistent with the proposed redox mechanism of NB in Figure 5B. All the electrochemical results suggest that Mn-CPM-99/ITO has better response sensitivity to NB and it is more suitable as an electrochemical sensor for detecting NB in aqueous solution.

### 3.4 Amperometric (i-t) determination of NB on Mn-CPM-99/ITO

**FIGURE 7:**
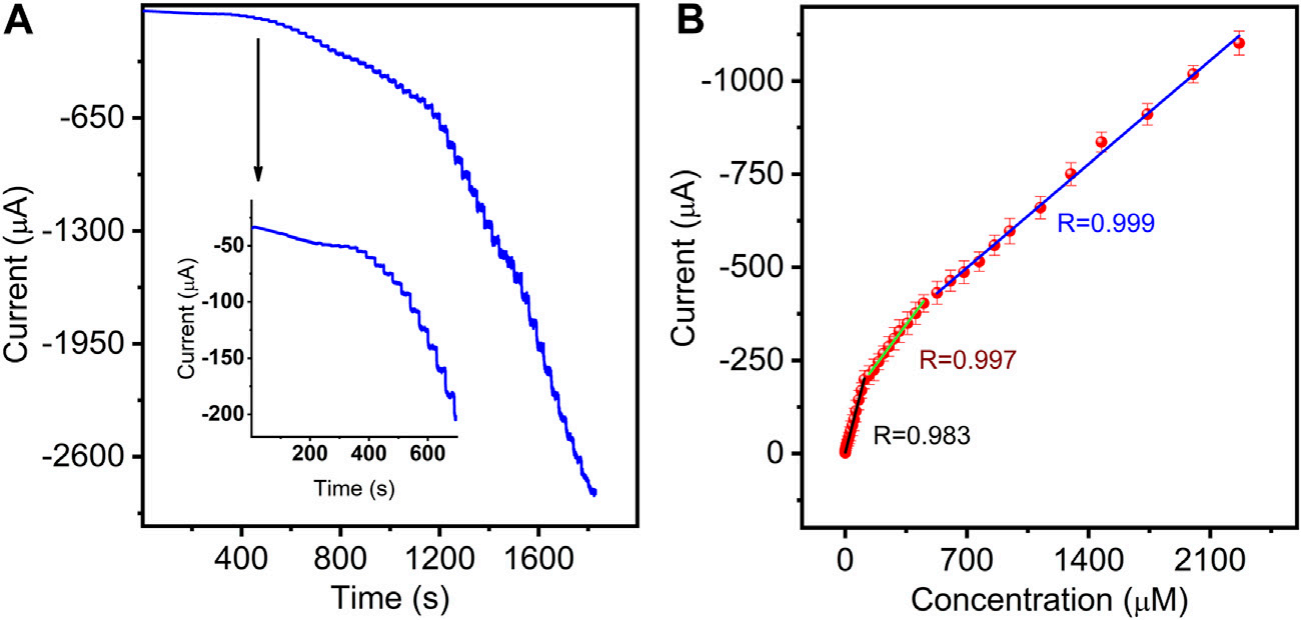
**(A)** Amperometric responses of the sensor in 3.0 mL stirring 0.4 M NaCl aqueous solution with successive additions of NB (0.005–2266 μM). **(B)** Corresponding calibration curves between electrocatalytic currents and NB concentrations (error bars represent standard deviations for three tests).

To obtain higher response sensitivity, the chronoamperometric technique was performed. As shown in Figure 7A, upon successive addition of different concentrations of NB into stirred solution, the sensor exhibited rapid and typical amperometric current response to NB at the applied potential of −0.77 V. The inset of Figure 7A shows a magnified plot of the reduced current response at low NB concentration. The reduction peak currents grew linearly with the increase in three NB concentration ranges from 5 nM to 109.5 μM, 136.7–450.2 μM, and 527.5–2266 μM (Figure 7B) with the corresponding sensitivities of 1.817 (R = 0.983), 0.621 (R = 0.997), and 0.398 μA μM^−1^ (R = 0.999), respectively. Obviously, at low concentration, the sensor showed higher sensitivity for NB determination due to more available catalytic sites. The limit of detection (LOD) was calculated as low as 1.3 nM (Wu et al., 2018), which is lower than the permissible limit in water (16.25 μM) regulated by APHA (Li et al., 2023). The analytical performances are comparable with those in previously reported NB sensors (Supplementary Table S1).

### 3.5 Analytical performance of the proposed sensor

**FIGURE 8:**
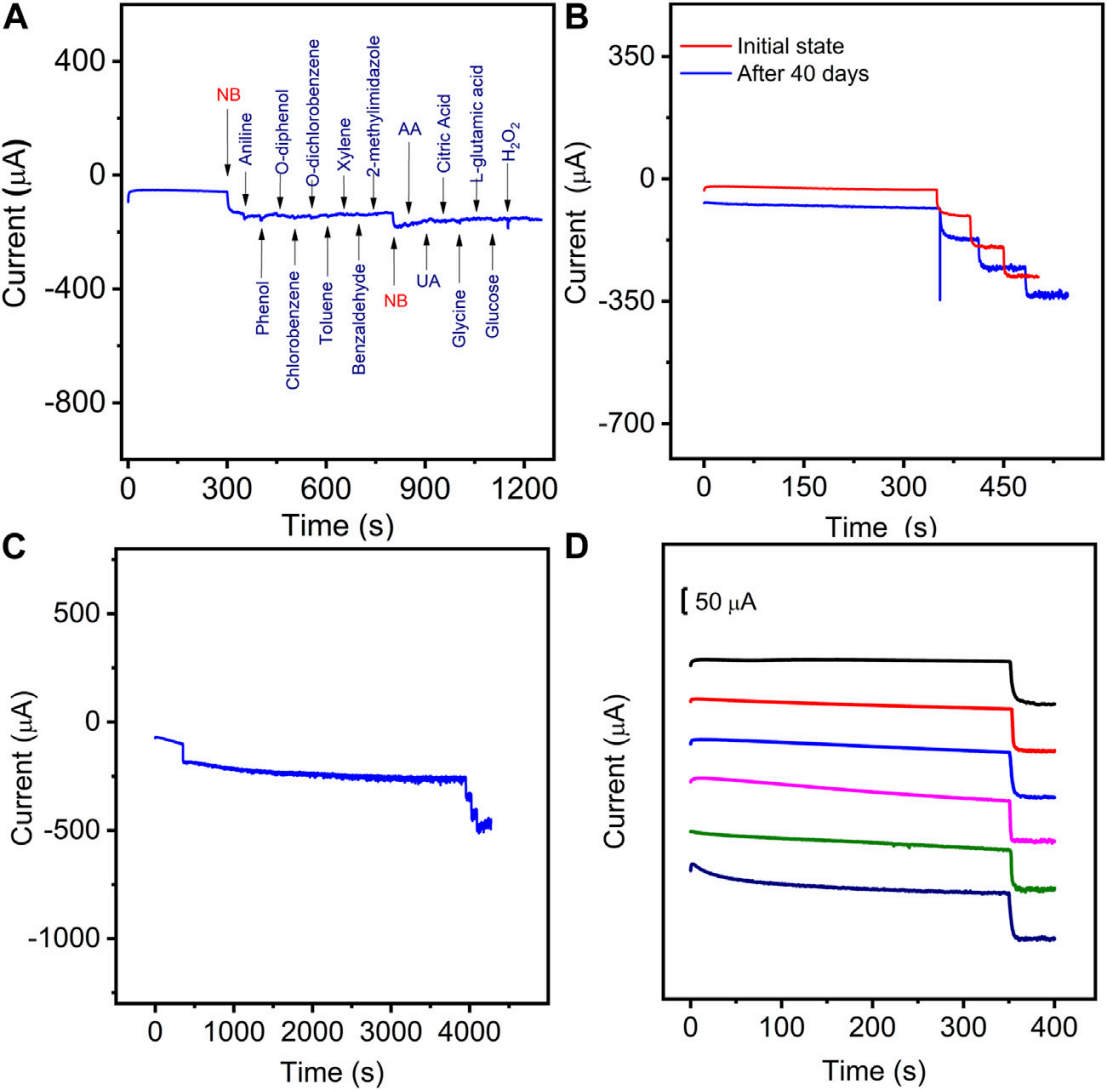
**(A)** Amperometric responses of the Mn-CPM-99/ITO sensor in 3.0 mL stirring 0.4 M NaCl aqueous solution with successive additions of 50.0 μM NB, 200 μM Aniline, phenol, o-diphenol, chlorobenzene, o-dichlorobenzene, Toluene, xylene, benzaldehyde, 2-Methylimidazole, AA, UA, citric acid, Glycine, L-glutamic acid, glucose, and H_2_O_2_ at the applied potential of −0.8V. **(B)** Storage stability of the Mn-CPM-99/ITO sensor in response to 50.0 μM NB after 40 days. **(C)** Successive current response of the sensor toward 50.0 μM NB before and after 4000 s response. **(D)** The current-time curves of six Mn-CPM-99/ITO electrodes toward 50 μM NB.

The selectivity of the presented sensor towards Nb was first evaluated by adding 4-fold interfering substances using the amperometric (*i-t*) method. As shown in Figure 8A, the current responses on NB were not interfered by the injection of the common organics (aniline, phenol, o-diphenol, chlorobenzene, o-dichlorobenzene, toluene, xylene, benzaldehyde, 2-methylimidazole), biomolecules (ascorbic acid (AA), uric acid (UA), citric acid, glycine (Gly), L-glutamic acid (L-GAA), glucose (Glu), and hydrogen peroxide (H_2_O_2_). Some common cations in aqueous solution were also evaluated including K^+^, Na^+^, Ca^2+^, Mg^2+^, Fe^2+^, Ni^2+^, Fe^3+^, and Cr^3+^ (Supplementary Figure S11). The current response of the interfering metal cations is obviously lower than that of NB at the same concentration level. These results indicate that the proposed sensor has good selectivity for NB detection.

The repeatability and stability reveal the potential capabilities of the proposed method for practical applications. The long-term storage stability of the sensor was evaluated by measuring the response currents of NB for 40 days of storage (Figure 8B), which showed that all the current responses towards three concentrations of NB retained over 97% of their original currents. In Figure 8C, the catalytic current of 50 μM NB does not decay for 4000 s of successive response, furthermore, the sensor still shows a sensitive response to NB, indicating good operational stability. The reproducibility of the sensor was evaluated by utilizing six modified electrodes on different GCEs. As shown in Figure 8D, the relative standard deviations were calculated to be 3.4%, indicating high reproducibility. Therefore, the satisfactory stability and reproducibility suggest that Mn-CPM-99/ITO is potentially applicable as a reliable sensor for the determination of NB in real water samples.

### 3.6 Analysis of real samples

The practicality of the Mn-CPM-99/ITO electrode was verified by the determination of NB in river water and vegetable (Pakchoi) samples. The Pakchoi vegetable was sprayed with 5 mL 20 mM NB solution. After 1 day, 3 g of fresh leaves were grinded and dissolved in 10 mL ethanol, which was then centrifuged at 6000 rpm. The obtained supernatant of vegetable and river water were filtered through 0.22 μm membrane filter and used directly for the actual sample analysis by standard addition method (Supplementary Figure S12). As shown in Table 1, NB in river water could not be detected while an average of 0.85 μM NB could be detected in the NB-pretreated vegetable samples. All of the recoveries with the added NB standard solution ranged from 98.8% to 101.8%, indicating that the electrochemical sensor based on Mn-CPM-99 MOF has good utility for the determination of NB content in real water or vegetables.

**TABLE 1:** Detection of NB in the real samples.

Samples	Added (µM)	Average detected value (µM)	Average recovery (%)	Mean relative standard deviation (%)
River water	−^a^	−	−	−
	3.00	3.01	100.2	3.17
	6.00	5.93	98.9	3.70
Pakchoi vegetable	−	0.85	−	4.39
	5.00	5.87	100.4	1.16
	10.00	11.03	101.8	0.70

^a^ NB, could not be detected.

## 4 Conclusion

In conclusion, a concave quadrangular bipyramidal Mn-CPM-99 MOF was successfully designed based on the coordination reaction between MnTCBPPCl organic ligands and Zr_6_ clusters. The structure and electrochemical properties of Mn-CPM-99 MOF were compared with those of Mn-PCN-222 MOF with rod-shaped structure (constructed form TCPP and Zr_6_). Due to the broadened chain of TCPP organic ligands by the phenyl group, Mn-CPM-99 MOF shows the bipyramidal structure with higher porosity and larger specific surface area than those of Mn-PCN-222. After comparing their morphology, electrochemical behavior, and electrocatalytic ability, it is found that Mn-CPM-99 modified ITO electrode has better electrocatalytic performances towards NB reduction. The high sensitivity and selectivity, low LOD, wide linear concentration range, and good reproducibility make the presented sensor (Mn-CPM-99/ITO) successfully applied to the detection of NB in river water and vegetable samples. Therefore, the strategy based on the structural design will broaden the application of the porphyrin-based MOF materials and provide a new platform to study the relationship between structural improvement and its function.

## Data Availability

The original contributions presented in the study are included in the article/Supplementary Material, further inquiries can be directed to the corresponding authors.
