# Supplementary material for: Construction of porphyrinic manganese-organic frameworks based on structural regulation for electrochemical determination of nitrobenzene in water and vegetable samples
Source: Front Chem. 2024 Mar 20;12:1380551. doi: 10.3389/fchem.2024.1380551 (PMC10987814; doi:10.3389/fchem.2024.1380551)
Supplement: Supplementary file 1 [file DataSheet1.docx]

**Supporting Information**

Construction of Porphyrinic Manganese-organic Frameworks Based on Structural Regulation and for Electrochemical Determination of Nitrobenzene in Water and Vegetable Samples

**Li Wang, Mengjie Zhang, Yuanyuan Li, Xiumei Chen, Hao Qin, Jin Yang, Suhua Fan*, Hai Wu***

Anhui Province Key Laboratory of Environmental Hormone and Reproduction, Anhui Province Key Laboratory for Degradation and Monitoring of Pollution of the Environment, Fuyang Normal University, Fuyang, Anhui 236037, PR China.

*** Correspondence:**

Suhua Fan; Hai Wu

E-mail address: [fansuhua@fudan.edu.cn](mailto:fansuhua@fudan.edu.cn); [wuhai317@126.com](mailto:ceszxy@mail.sysu.edu.cn).

**1. Synthesis of Mn-PCN-222 and Mn-CPM-99**

*1.1. The synthetic routes of Mn-TCPPCl and Mn-TCBPPCl*

**SCHEME S1.** The synthetic routes of MnTCPPCl and MnTCBPPCl

*1.2. Synthesis of MnTCPPCl Ligand*

*1.2.1. 5,10,15,20-Tetrakis(4-methoxycarbonylphenyl) porphyrin (H_2_TPPCOOCH_3_).*

Methyl p-formylbenzoate (6.9 g, 0.042 mol) was dissolved in propionic acid (100 mL) with stirring and then 3.0 mL pyrrole (0.043 mol) was added to the propionic acid solution and refluxed for 12 hours in the dark. After cooling and recrystallisation at room temperature, 1.28 g purple crystalline material was collected by diafiltration and recrystallization. ^1^H NMR was used to characterize the obtained precursor of H_2_TPPCOOCH_3_, which was shown in Figure S1. ^1^H NMR (400 MHz, Chloroform-*d*) δ 8.85 (s, 8H), 8.48 (d, *J* = 8.1 Hz, 8H), 8.33 (d, *J* = 8.1 Hz, 8H), 4.15 (s, 12H), -2.80 (s, 2H).


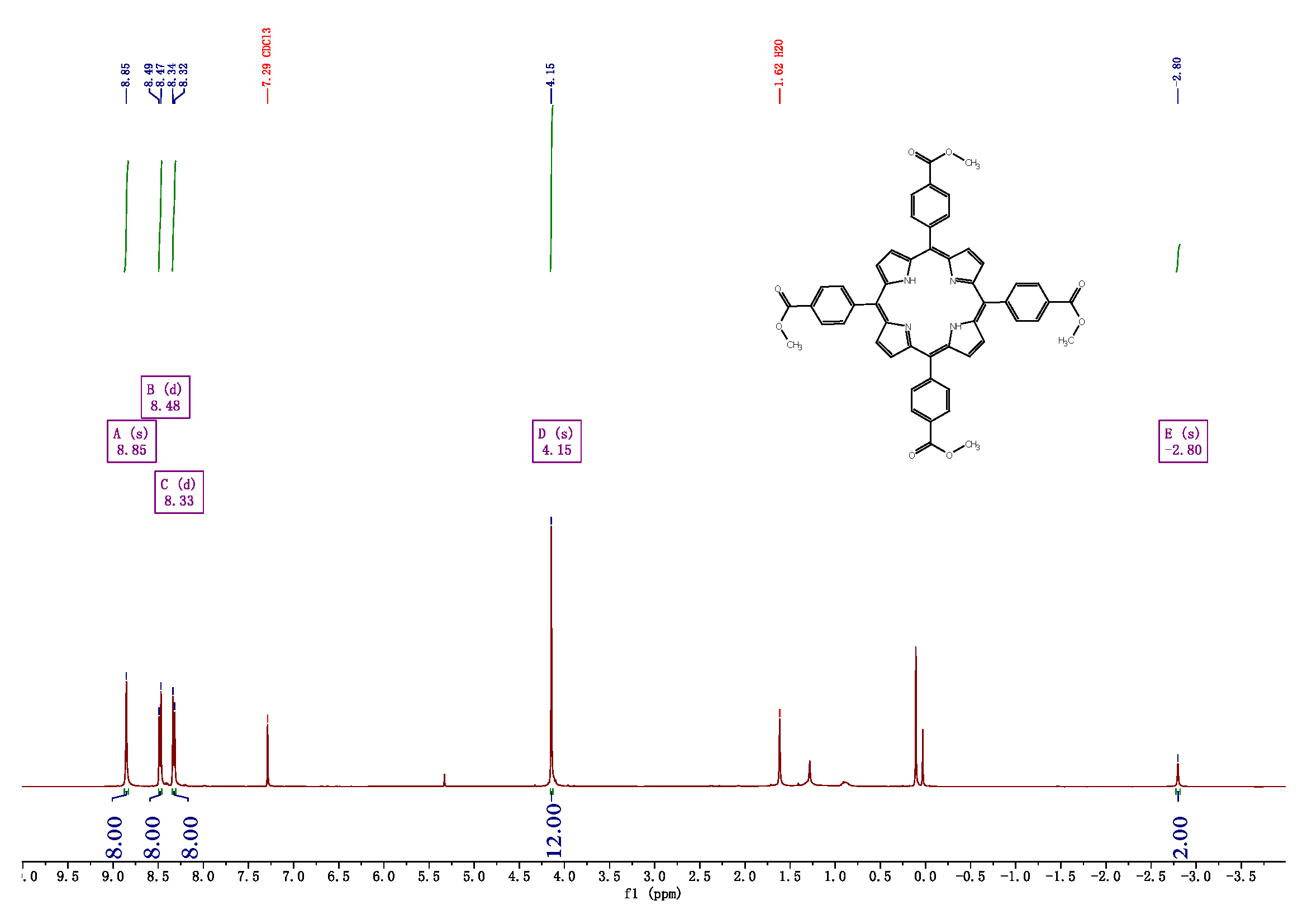


**FIGURE S1.** ^1^H NMR spectrum of 5,10,15,20-Tetrakis(4-methoxycarbonylphenyl) porphyrin

(H_2_**TPPCOOMe**) (400 MHz, CDCl_3_)

*1.2.2. [5,10,15,20-Tetrakis(4-methoxycarbonylphenyl) porphyrinato]-Mn (III) Chloride (MnTPPCOOMeCl)*

0.847 g H_2_TPPCOOCH_3_ (1.0 mmol) and 2.5 g MnCl_2_•4H_2_O (20 mmol) were dissolved in DMF (100 mL) with stirring and refluxed for 6 hours in the dark. After cooling to room temperature, the precipitate was washed and filtered with water for 3 times. The obtained solid of MnTPPCOOMeCl was dissolved in DMF again and water was added to remove the inorganic salt, which was repeated three times. After spin-drying, the purified green MnTPPCOOMeCl was obtained.

*1.2.3. [5,10,15,20-Tetrakis(4-carboxyphenyl) porphyrinato]-Mn (III) Chloride (MnTCPPCl).*

0.75 g MnTPPCOOMeCl was dissolved in 25 mL THF and 25 mL MeOH, then 25 mL 1.88 M KOH aqueous solution was added to the mixture with stirring, which was refluxed at 85 °C for 12 hours in the dark. After cooling to room temperature, the organic solvent was evaporated and the obtained solid was completely dissolved in water with heating, then the homogeneous solution was acidified by 1 M HCl (pH=2). The carboxylated MnTCPPCl ligand was obtained by filtration through water washing and dried under vacuum.

*1.3. Synthesis of MnTCBPPCl Ligand*

*1.3.1. 4'-Formyl-biphenyl-4-carboxylic acid ethyl ester (FBPCEt)*

ethyl 4-bromobenzoate (1.26 g, 0.0055 mol) and 4-formylphenylboronic acid (0.99 g, 0.0066 mol) was dissolved in Toluene (60 mL) and THF (40 mL) mixed solvent and 2 drops of methyltri-octylammonium chloride was added to the mixture, which was evacuated and filled with nitrogen. The mixed solution of K_2_CO_3_ (13.8 g, 100 mmol) and 4-(triphenylphosphine) palladium (1.2 g, 1 mmol) was added and stirred thoroughly at 85°C for 12 h. After cooling to room temperature under vacuum, the organic matter was extracted with water and DCM for three times. After spin-drying, the target product (FBPCEt) was purified and separated by using chromatography (DCM:PE=1:1). Figure S2. ^1^H NMR (400 MHz, Chloroform-*d*) δ 10.06 (s, 1H), 8.14 (d, *J* = 8.5 Hz, 2H), 7.97 (d, *J* = 8.3 Hz, 2H), 7.77 (d, *J* = 8.2 Hz, 2H), 7.69 (d, *J* = 8.5 Hz, 2H), 4.40 (q, *J* = 7.1 Hz, 2H), 1.41 (t, *J* = 7.2 Hz, 3H).


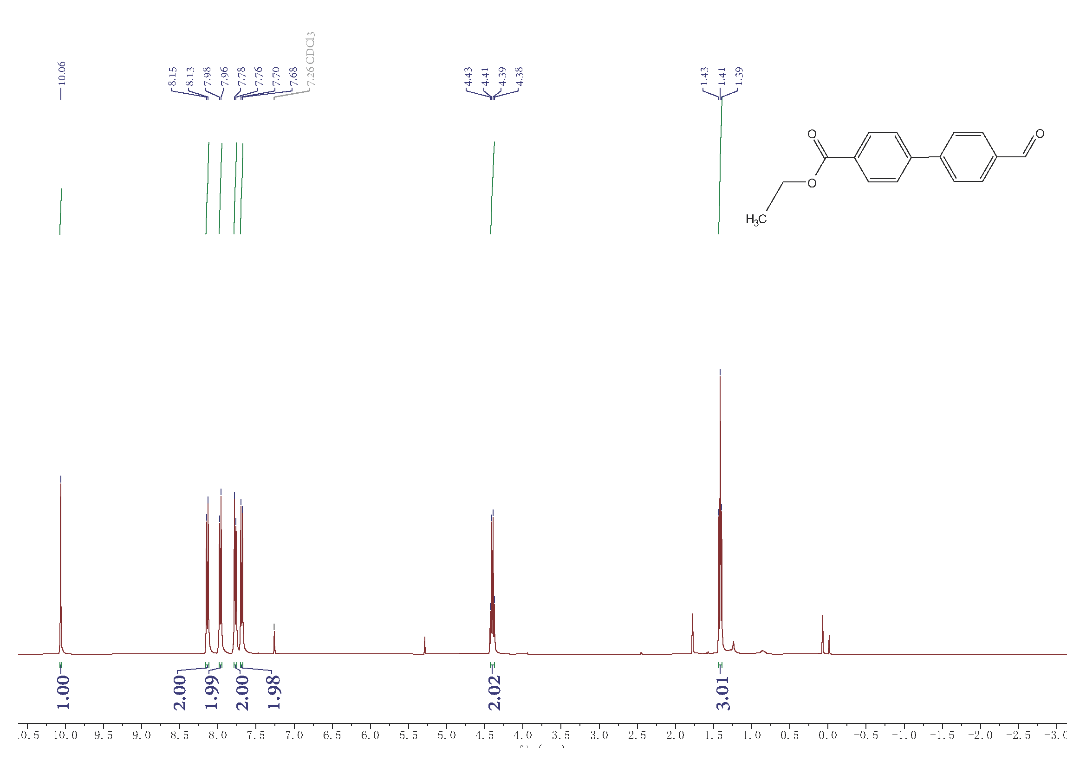


**FIGURE S2.** ^1^H NMR spectrum of 4'-formyl-biphenyl-4-carboxylic acid ethyl ester

(**FBPCEt**) (400 MHz, CDCl_3_)

*1.3.2. the synthesis of 5,10,15,20-Tetrakis(4-ethylcarboxy**biphenyl) porphyrin (H_2_TEtCBPP)*

Ethyl 4'-formyl-biphenyl-4-carboxylate (FBPCEt, 10.68 g, 0.042 mol) was added to propionic acid (100 ml) with stirring and refluxed. Pyrrole (3.0 ml, 0.043 mol) was added to the refluxed propionic acid and was continually refluxed in the dark for 3 hours. After cooling to room temperature, the purple material was collected by diafiltration and recrystallization. The resulting crude product was chromatographed on silica gel (DCM/CH_3_OH=300/1) to give the desired product (H_2_TEtCBPP). Figure S3. ^1^H NMR (400 MHz, Chloroform-*d*) δ 8.94 (s, 8H), 8.29 (dd, *J* = 15.3, 7.9 Hz, 16H), 8.00 (dd, *J* = 12.4, 7.9 Hz, 16H), 4.48 (q, *J* = 7.1 Hz, 8H), 1.48 (t, *J* = 7.1 Hz, 12H), -2.68 (s, 2H).


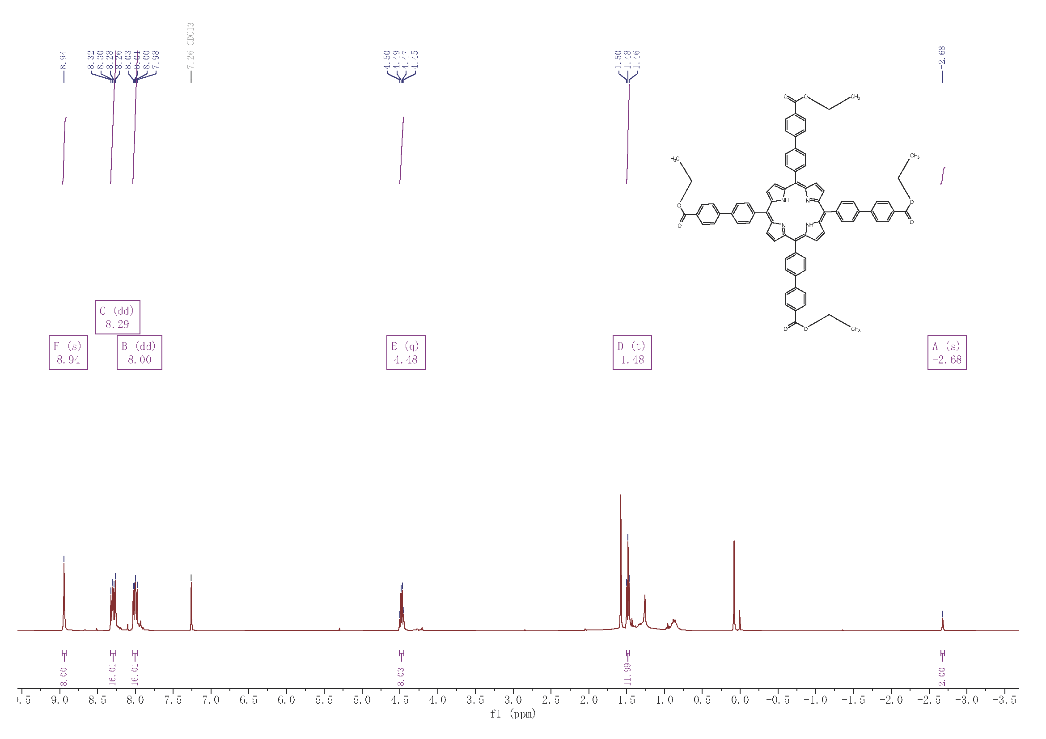


**FIGURE S3.** ^1^H NMR spectrum of 5,10,15,20-tetrakis(4-ethylcarboxybiphenyl) porphyrin

(H_2_**TEtCBPP**) (400 MHz, CDCl_3_)

*1.3.3. [5,10,15,20-Tetrakis(4-ethylcarboxybiphenyl) porphyrin]-Mn (III) Chloride (MnTEtCBPPCl).*

1.21 g H_2_TEtCBPP (1.0 mmol) and 2.5 g MnCl_2_⋅4H_2_O (20 mmol) were dissolved in DMF (100 mL) with stirring and refluxed for 6 hours in the dark. After cooling to room temperature, the precipitate was washed and filtered with water for 3 times. The obtained solid of MnTEtCBPPCl was dissolved in DMF again and water was added to remove the inorganic salt, which was repeated three times. After spin-drying, the purified green MnTEtCBPPCl was obtained.

*1.3.4. [5,10,15,20-Tetrakis(4-carboxybiphenyl) porphyrin]-Mn (III) Chloride (MnTCBPPCl).*

1.0 g MnTEtCBPPCl was dissolved in 25 mL THF and 25 mL MeOH, then 25 mL 1.88 M KOH aqueous solution was added to the mixture with stirring, which was refluxed at 85 °C for 12 hours in the dark. After cooling to room temperature, the organic solvent was evaporated and the obtained solid was completely dissolved in water with heating, then the homogeneous solution was acidified by 1 M HCl (pH=2). The carboxylated MnTCBPPCl ligand was obtained by filtration through water washing and dried under vacuum.

**2. Characterization for the Mn-PCN-222 and Mn-CPM-99 MOFs**


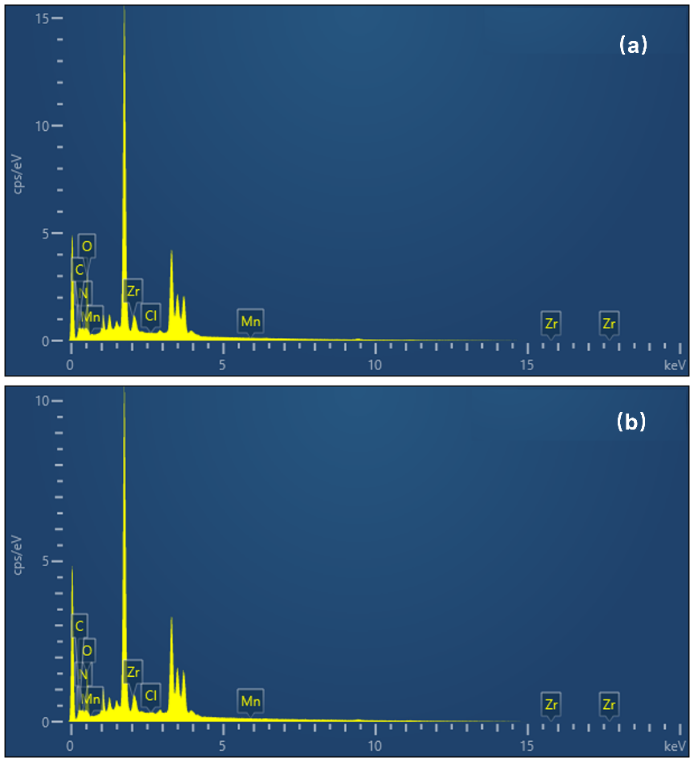


**FIGURE S4.** EDS of (a)Mn-PCN-222 and (b)Mn-CPM-99.


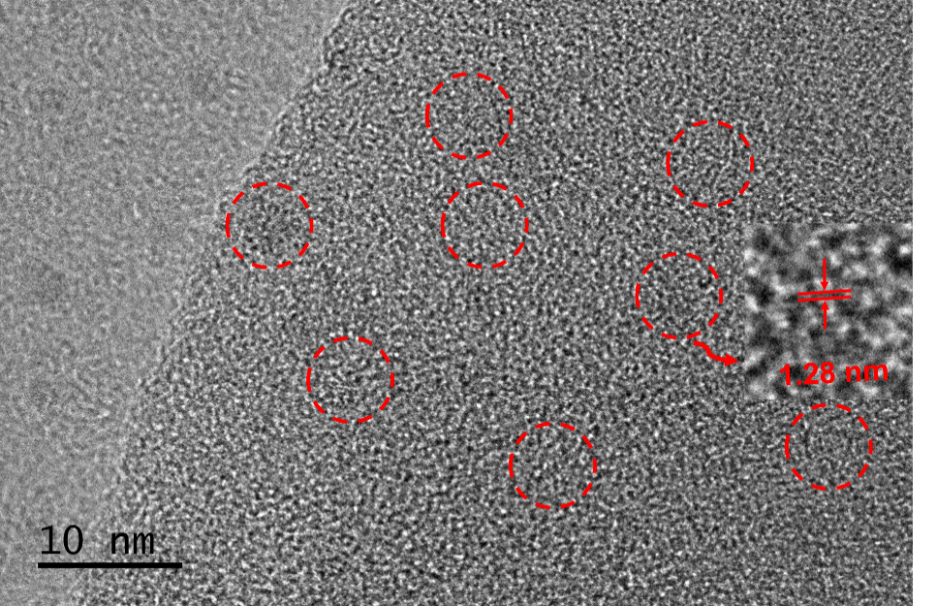


**FIGURE S5.** HRTEM images of the Mn-CPM-99.





**FIGURE S6.** SAED pattern of the Mn-CPM-99.


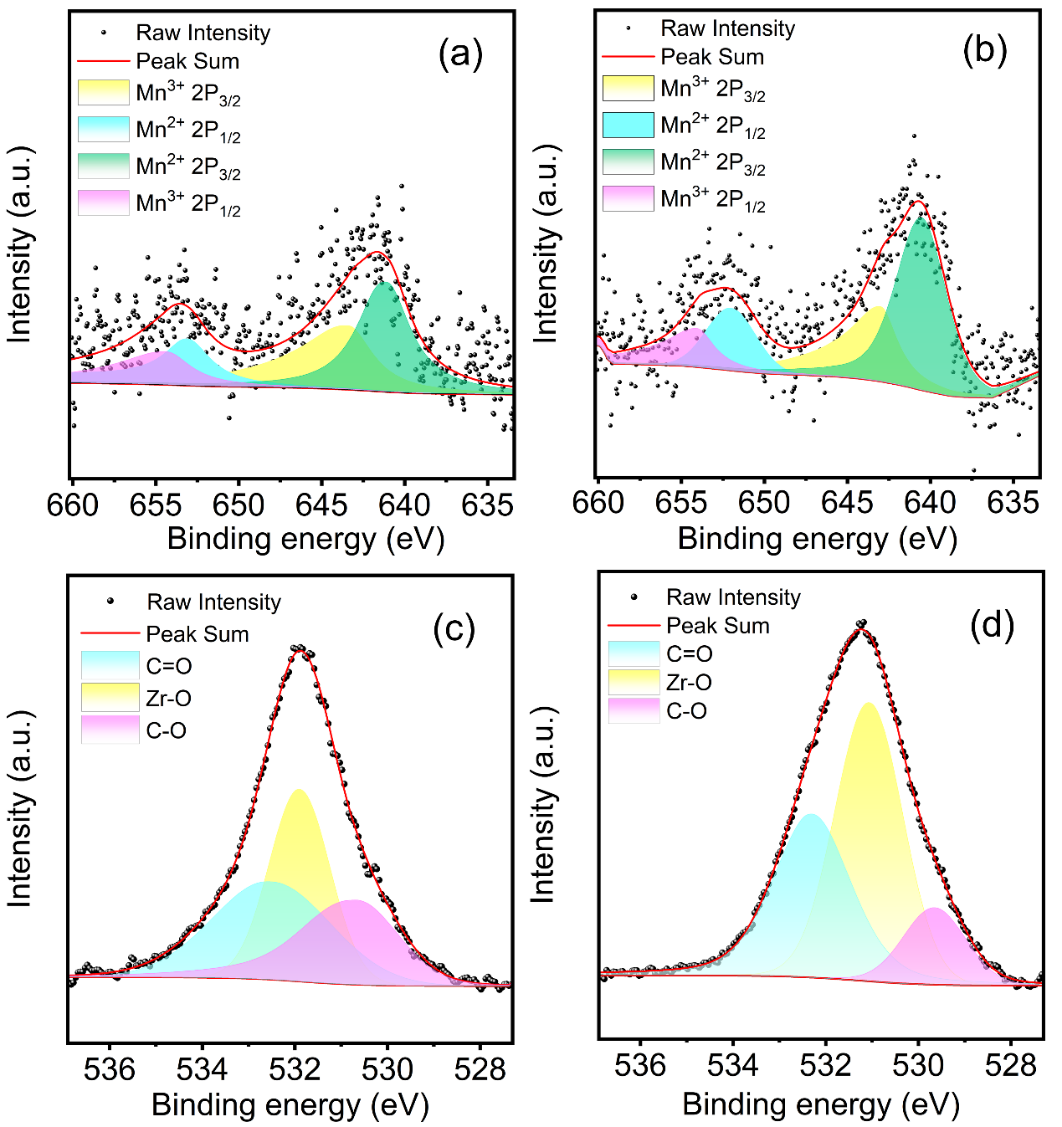


**FIGURE S7.** The high resolution XPS spectra of Mn 2P, O 1s spectra of Mn-PCN-222 (a, c) and Mn-CPM-99 (b, d).

**3. Active surface area of the modified electrodes**

According to the Randles-Sevcik equation (1) (Karthik et al., 2023; Sukanya et al., 2018), the active electrochemical area of different modified electrodes can be calculated:

*I*_p_=2.69×10^5^ n^3/2^ D^1/2^ v^1/2^ A c (1)

where *c* is the concentration of [Fe(CN)_6_]^3−/4−^ (mol⋅cm^−3^), *A* represents the electrochemically active surface area (cm^2^), *v* is the scan rate (*v*⋅*s*^−1^), D is the diffusion coefficient of [Fe(CN)_6_]^3−/4−^ (7.6×10^−6^ cm^2^⋅s^−1^), n is the number of electrons transferred in the redox reaction, and *I*_p_ is the redox peak current. From Eq. (1), the electrochemically active surface areas of ITO, Mn-PCN-222/ITO and Mn-CPM-99/ITO were calculated to be 0.353, 0.497, and 0.558 cm^2^, respectively. And the calculated active surface area of Mn-CPM-99/ITO was significantly larger than that of ITO or Mn-PCN-222/ITO, indicating that the composite has a larger electrically active surface area, thus providing a more efficient electrochemical sensor.

**
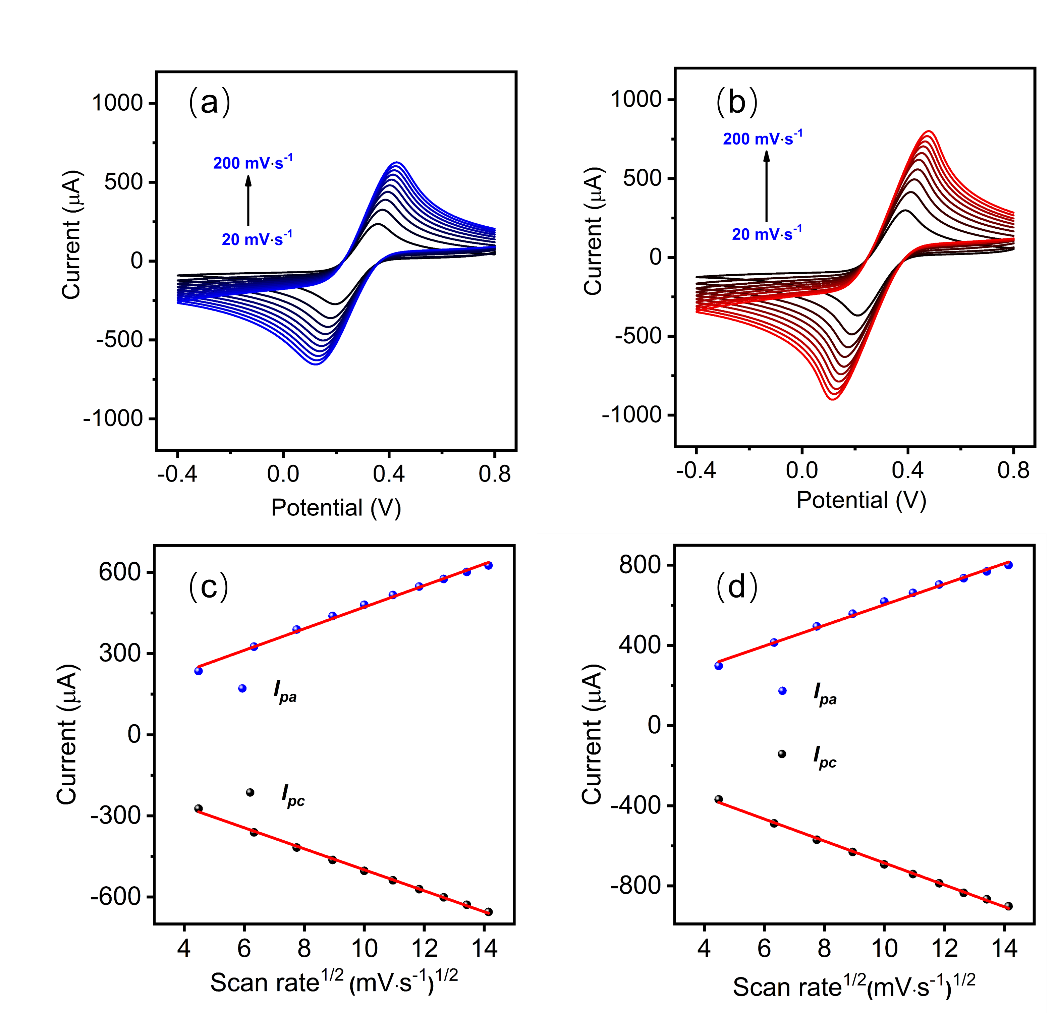
**

**FIGURE S8.** CVs of different scan rates from 20 to 200 mV⋅s^−1^ on (a) ITO, (b) PCN/ITO recorded in 0.005 M [Fe(CN)_6_]^3−/4−^ containing 0.1 M KCl. (c), (d) The linear plot for *I*_pa_, *I*_pc_ against the square root of the scan rate (ITO, PCN/ITO).

4. **The electrocatalysis of NB on MnTCPP/ITO and MnTCBPP/ITO electrodes**

To investigate inherent redox activity of the ligands and Mn(III)/Mn(II) couple in the Mn-PCN-222 and Mn-CPM-99 structure, we used TCPP, TCBPP, MnTCPP, and MnTCBPP to modify ITO electrode investigate their electrocatalytic activity. As shown in Figure S9, in the absence of nitrobenzene (NB), TCPP and TCBPP show the inherent electrochemical property of porphyrin ligands. MnTCPP and MnTCBPP also show irreversible redox process and weak redox peaks of Mn(III)/Mn(II). This phenomenon may be caused by the hydrophobicity of the structure of the metalloporphyrin derivatives, which makes it difficult to show the electrochemical behavior of Mn(III)/Mn(II) couple at the solid-liquid interface. In the presence of NB, both types of modified electrodes show good catalytic performances (Figure S4), which are better than that of bare ITO (Figure 4e in manuscript). Furthermore, the MnTCBPP/ITO electrode exhibits better catalytic ability than that of MnTCPP/ITO. Therefore, the inherent redox activity of the Mn(III)/Mn(II) couple in the Mn-PCN-222 and Mn-CPM-99 structure can be acted as electron intermediate for accelerating the electron transport (Wu et al., 2014). The results are consistent with those of Mn-PCN-222 and Mn-CPM-99 modified electrodes and the results in reported reference (Zhou et al., 2021; Lieske et al., 2019; Mourzina et al., 2020).


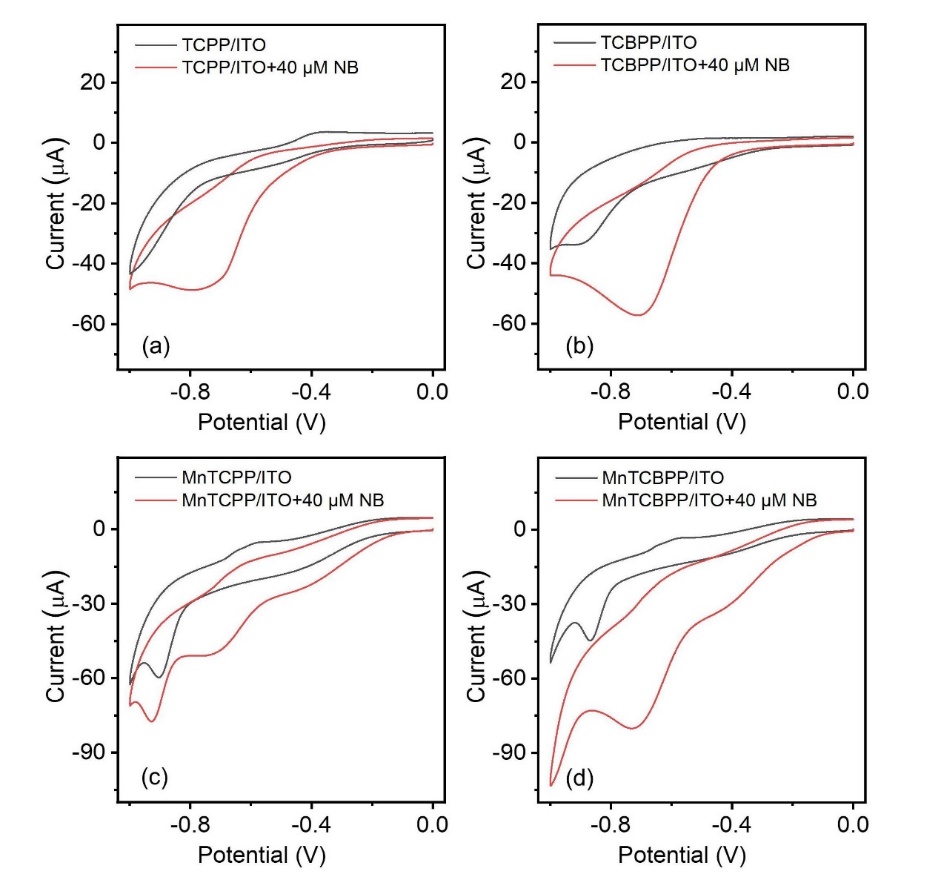


**FIGURE S9.** CVs of TCPP/ITO (a), TCBPP/ITO (b), MnTCPP/ITO (c), and MnTCBPP/ITO (d) in 0.4 M NaCl (pH 7.0) without and with 40 μM NB, respectively.

**4. Condition optimization of electrolyte concentration**

**
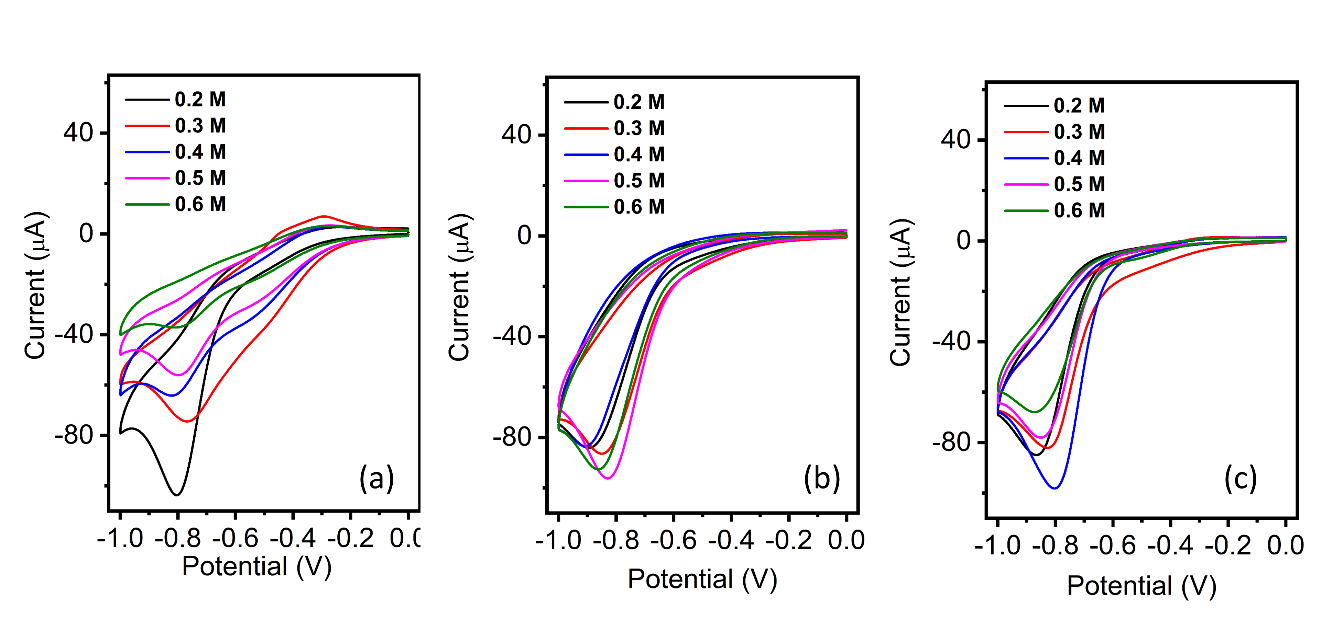
**

**FIGURE S10.** CVs of ITO (a), Mn-PCN-222/ITO (b), and Mn-CPM-99/ITO (c) at different concentrations of NaCl from 0.2 to 0.6 M in presence of 40 μM NB.

**5. Selectivity of the presented sensor for metal ions**

**
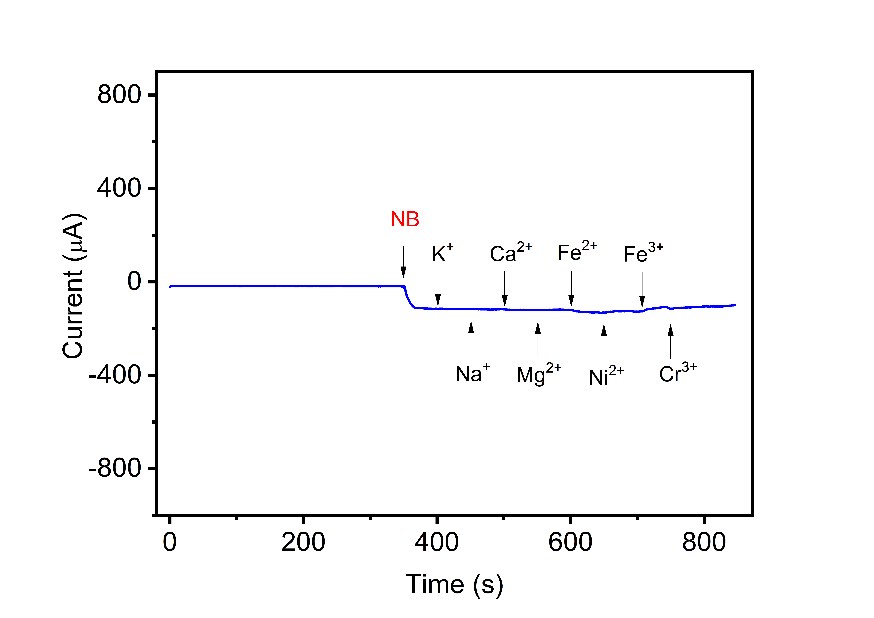
**

**FIGURE S11.** Amperometric responses of the sensor in 3.0 mL stirring 0.4 M NaCl (pH 7.0) aqueous solution with successive additions of 50.0 μM nitrobenzene, 50 μM K^+^, Na^+^, Ca^2+^, Mg^2+^, Fe^2+^, Ni^2+^, Fe^3+^, and Cr^3+^ at the applied potential of −0.8V.

**6. Detection of NB in real samples**


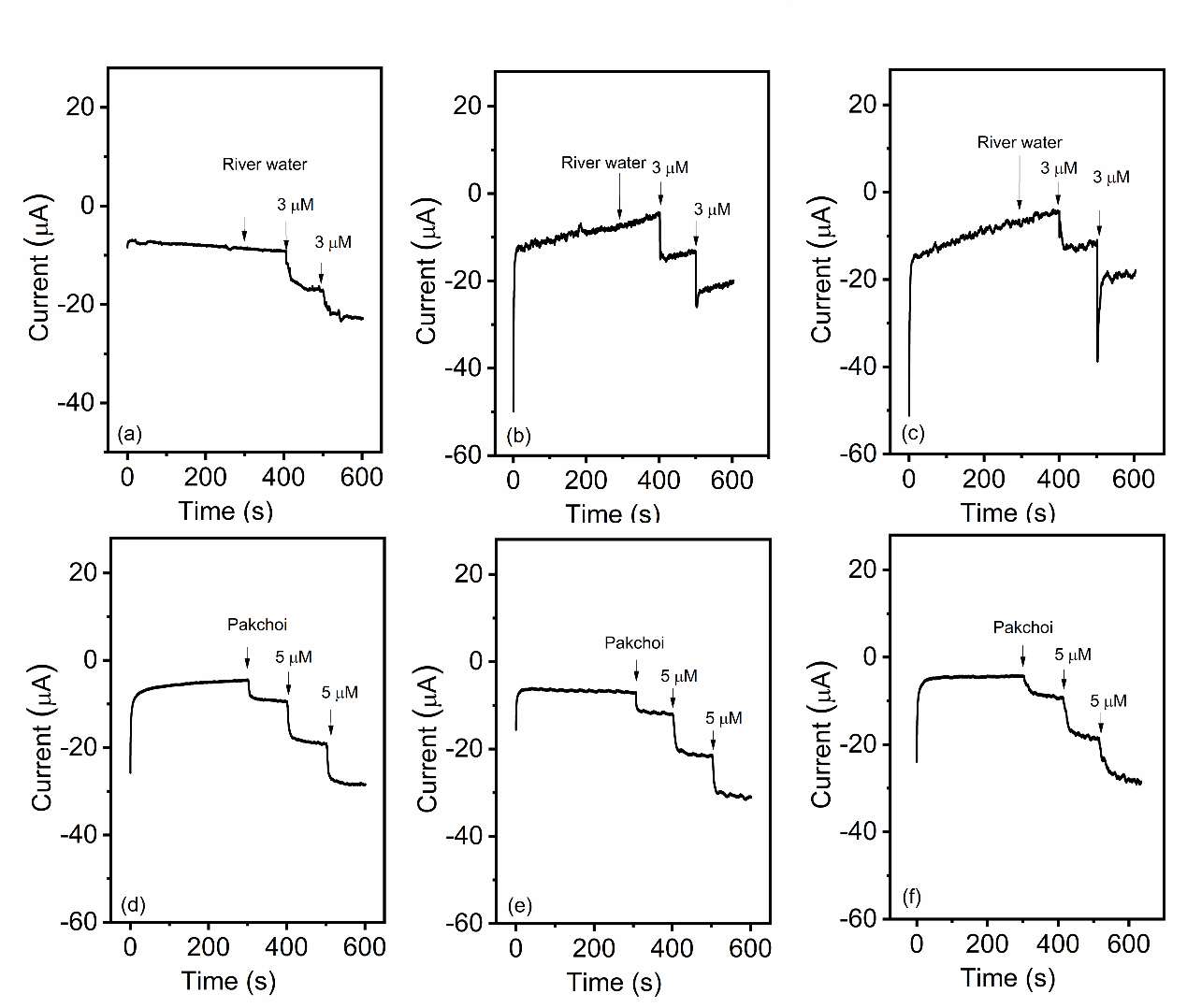


**FIGURE S12.** Amperometric responses of the sensor in 3.0 mL stirring 0.4 M NaCl (pH 7.0) aqueous solution toward river water (a-c) and NB-pretreated Pakchoi vegetable (d-f) samples by using standard addition method.

**7. Comparison for the analytical performances between the presented sensor and reported sensor**

**Table S1.** Comparison of the performance of the proposed electrode with other reported electrochemical NB sensors

| **Modified Electrode** | | **Technique** | **Linear Range (μM)** | **LOD**  **(μM)** | **Ref.** |
| --- | --- | --- | --- | --- | --- |
| MMPCMs/GCE | LSV | | 0.2 - 40 | 0.008 | (Ma et al., 2012) |
| OMC/DDAB/GCE | LSV | | 20 - 2900 | 10 | (Qi et al., 2008) |
| Ag/ATPNPs /GCE | LSV | | Up to 30 | 1.1 | (Liang et al., 2011) |
| SANb-BCN/GCE | LSV | | 2 - 100,  100 - 600 | 0.7 | (Li et al., 2021) |
| Hanging mercury drop electrode | LSV | | 14.7 - 1000 | 5 | (Liang et al., 2007) |
| PNMPC/Nafion/GC | LSV | | 1 - 200 | 0.05 | (Zhang et al., 2012) |
| ZSO-CN/GCE | LSV | | 30 - 100,  100 - 1000 | 2.2 | (Vinoth et al., 2020) |
| β-CD /GO/SPCE | LSV | | 0.05 - 447 | 0.021 | (Balasubramanian et al., 2019) |
| β-CD1.2mg/GO/SPCE | LSV | | 0.5 - 100,  100 - 1000 | 0.184 | (Velmurugan et al., 2017) |
| GO/MgO/GCE | DPV | | 0.1 - 2333.5 | 0.01 | (Kokulnathan et al., 2021) |
| Ni/Fe(SDS)-LDH | DPV | | 1 - 10, 10 -100, 100 - 350 | 0.093 | (Li et al., 2021) |
| AuNPs/GCE | DPV | | 0.1 - 600 | 0.016 | (Emmanuel et al., 2014) |
| CeO_2_ NPs | DPV | | 0.1 - 520 | 0.092 | (Sangili et al., 2018) |
| Pd-GG-g-PAM-silica/GCE | DPV | | 1 - 1900  1900 - 3900 | 0.06 | (Rastogi et al., 2014) |
| NaOH/GCE | DPV | | 0.1 - 1000 | 0.04 | (Zhang et al., 2007) |
| PCN-222(Fe)/p3HT-p3TPA | DPV | | 0.05 - 1.00, 1.00- 100 | 0.047 | (Li et al., 2023) |
| SNPs/GCE | DPV | | 5 - 40 | 0.027 | (Shivakumar et al., 2020) |
| MWCNTs/GCE | CV | | 0.08 - 32.5 | 0.024 | (Fakhari et al., 2011) |
| SiO_2_/Au/GC | CV | | 0.1 - 25 | 0.1 | (Singh et al., 2012) |
| TiO_2_GO | CV | | 2 - 8 | 2.64 | (Ruiz-Ramirez et al., 2021) |
| SSG-AuAg | SWVb | | 1 - 80 | 0.045 | (Manivannan et al., 2018) |
| Ag/SiO_2_ NPs | SWV | | 5 - 45 | 0.5 | (Rameshkumar et al., 2014) |
| BiF/CPE | SWV | | 1 - 100 | 0.83 | (Luo et al., 2010) |
| CuS-BCN/GCE | SWV | | 0.5 - 150, 150-1000 | 0.12 | (Yuan et al., 2023) |
| C_60_/CPE | SDCV | | 50 - 6000 | 30 | (Qian et al., 1997) |
| SMO/N-rGO | Amperometry | | 0.0071 - 1001.9, 1108 - 2578 | 0.0025 | (Karthik et al., 2023) |
| PAA -AgNPs/GCE | Amperometry | | 10 - 600 | 1.68 | (Kariuki et al., 2016) |
| EDAS-AgNPs/GCE | Amperometry | | 0.025 - 10 | 0.025 | (Maduraiveeran et al., 2009) |
| PEDOT/MWCNT/CPE | Amperometry | | 0.25 - 43 | 0.083 | (Xu et al., 2014) |
| RGO–AgNPs/GCE | Amperometry | | 0.5 - 900 | 0.261 | (Karuppiah et al., 2015) |
| Au MOF-5 | Amperometry | | 20 – 500,  500 - 6000 | 15.3 | (Yadav et al., 2016) |
| **Mn-CPM-99/ITO** | **Amperometry** | | **0.005 - 109.5, 136.75 - 450.25, 527.5 - 2266** | **0.0013** | **This work** |

**References**

Balasubramanian, P., Velmurugan, M., Chen, S. M., Chen, T. W., Ye, Y. T. A (2019). single-step electrochemical preparation of cadmium sulfide anchored erGO/β-CD modified screen-printed carbon electrode for sensitive and selective detection of nitrite. *J. Electrochem. Soc.* 166, B690. doi: [10.1149/2.0981908jes](https://www.x-mol.com/paperRedirect/1518604945666727936).

Emmanuel, R., Karuppiah, C., Chen, S. M., Palanisamy, S., Padmavathy, S., Prakash, P. (2014). Green synthesis of gold nanoparticles for trace level detection of a hazardous pollutant (nitrobenzene) causing Methemoglobinaemia. *J. Hazard. Mater.* 279, 117-124. doi: [10.1016/j.jhazmat.2014.06.066](https://www.x-mol.com/paperRedirect/1212899865678127120).

Fakhari, A. R., Ahmar, H. (2011). A new method based on headspace adsorptive accumulation using a carboxylated multi-walled carbon nanotubes modified electrode: application for trace determination of nitrobenzene and nitrotoluene in water and wastewater. *Anal. Methods.* 3, 2593-2598. doi: [10.1039/c1ay05302g](https://www.x-mol.com/paperRedirect/1135878).

Kariuki, V. M., Fasih-Ahmad, S. A., Osonga, F. J., Sadik. O. A. (2016). An electrochemical sensor for nitrobenzene using π-conjugated polymer-embedded nanosilver. *Analyst* 141, 2259-2269. doi: [10.1039/c6an00029k](https://www.x-mol.com/paperRedirect/87289).

Karthik, R., Chavan, P. R., Sukanya, R., Dhakal, G., Shim, J. J., Breslin, C. B. (2023). Flower-like strontium molybdate anchored on 3D N-rich reduced graphene oxide aerogel composite: An efficient catalyst for the detection of lethal pollutant nitrobenzene in water samples. *Compos. Part. B-Eng.* 256, 110649. doi: [10.1016/j.compositesb.2023.110649](https://www.x-mol.com/paperRedirect/1630731719331622912).

Karuppiah, C., Muthupandi, K., Chen, S. M., Ali, M. A., Palanisamy, S., Rajan, A., et al. (2015). Green synthesized silver nanoparticles decorated on reduced graphene oxide for enhanced electrochemical sensing of nitrobenzene in waste water samples. *RSC Adv.* 5, 31139-31146. doi: [10.1039/c5ra00992h](https://www.x-mol.com/paperRedirect/1031544).

Kokulnathan, T., Jothi, A. I., Chen, S. M., Almutairi, G., Ahmed, F., Arshi, N., et al. (2021). Integrating graphene oxide with magnesium oxide nanoparticles for electrochemical detection of nitrobenzene. *J. Environ. Chem. Eng.* 9, 106310. doi: [10.1016/j.jece.2021.106310](https://www.x-mol.com/paperRedirect/1433539430195118080).

Li, M., Peng, X., Liu, X., Wang, H., Zhang, S., Hu, G. (2021). Single-atom niobium doped BCN nanotubes for highly sensitive electrochemical detection of nitrobenzene. *RSC Adv.* 11, 28988-28995. doi: [10.1039/d1ra05517h](https://www.x-mol.com/paperRedirect/1432847273389965312).

Li, S. S., Fang, J. H., Li, L., Zhu, M., Zhang, F., Zhang, B.Y., et al. (2021). An ultra-sensitive electrochemical sensor of Ni/Fe-LDH toward nitrobenzene with the assistance of surface functionalization engineering. *Talanta* 225, 122087. doi: [10.1016/j.talanta.2021.122087](https://www.x-mol.com/paperRedirect/1347336288115773440).

Li, Y.P., Zhuge, R. X., Zhang, T. (2023). MOF-Conductive Polymer Composite Electrode as Electrochemical Sensor of Nitrobenzene. *Inorg. Chem. Commun.* 154, 110904. doi: [10.1016/j.inoche.2023.110904](https://www.x-mol.com/paperRedirect/1665154693140824064).

Liang, F., Liu, B., Deng, Y., Yang, S., Sun, C. (2011). Preparation and characterization of attapulgite-silver nanocomposites, and their application to the electrochemical determination of nitrobenzene. *Microchim. Acta* 174, 407-412. doi: [10.1007/s00604-011-0647-7](https://www.x-mol.com/paperRedirect/1225026981588418563).

Liang, S. X., Zhang, H. K., Lu, D. (2007). Determination of nitrobenzene in wastewater using a hanging mercury drop electrode. *Environ. Monit. Assess.* 129, 331-337. doi: [10.1007/s10661-006-9366-7](https://www.x-mol.com/paperRedirect/1212984592229539845).

Lieske, L. E., Hooe, S. L., Nichols, A. W., Machan, C. W. (2019). Electrocatalytic reduction of dioxygen by Mn (III) meso-tetra (N-methylpyridinium-4-yl) porphyrin in universal buffer. *Dalton Trans.* 48, 8633-8641. doi:10.1039/C9DT01436E.

Luo, L., Wang, X., Ding, Y., Li, Q., Jia, J., Deng, D. (2010). Electrochemical determination of nitrobenzene using bismuth-film modified carbon paste electrode in the presence of cetyltrimethylammonium bromide. *Anal. Methods* 2, 1095-1100. doi: [10.1039/c0ay00150c](https://www.x-mol.com/paperRedirect/1137340).

Ma, J., Zhang, Y., Zhang, X., Zhu, G., Liu, B., Chen, J. (2012). Sensitive electrochemical detection of nitrobenzene based on macro-/meso-porous carbon materials modified glassy carbon electrode. *Talanta* 88 (2012) 696-700. doi: [10.1016/j.talanta.2011.11.067](https://www.x-mol.com/paperRedirect/1212926342859661330)

Maduraiveeran, G., Ramaraj, R. (2009). Potential sensing platform of silver nanoparticles embedded in functionalized silicate shell for nitroaromatic compounds. *Anal. Chem.* 81, 7552-7560. doi: [10.1021/ac900781d](https://www.x-mol.com/paperRedirect/1470857).

Manivannan, S., Jeong, J., Kang, D. K., Kim, K. (2018). One-step synthesis of AuAg alloy nanodots and its electrochemical studies towards nitrobenzene reduction and sensing. *Electroanal.* 30, 57-66. doi: [10.1002/elan.201700451](https://www.x-mol.com/paperRedirect/433261).

Mourzina, Y. G., Offenhäusser, A. (2020). Electrochemical properties and biomimetic activity of water-soluble meso-substituted Mn (III) porphyrin complexes in the electrocatalytic reduction of hydrogen peroxide. *J. Electroanal. Chem.* 866, 114159. doi: 10.1016/j.jelechem.2020.114159.

Qi, B., Lin, F., Bai, J., Liu, L., Guo. L. (2008). An ordered mesoporous carbon/didodecyldimethylammonium bromide composite and its application in the electro-catalytic reduction of nitrobenzene. *Mater. Lett.* 62, 3670-3672. doi: [10.1016/j.matlet.2008.04.027](https://www.x-mol.com/paperRedirect/1410646192738324480).

Qian, H., Ye, J., Jin, L. (1997). Study of the electrochemical properties of C_60_ modified carbon paste electrode and its application for nitrobenzene quantitation based on electrocatalytic reduction. *Anal. Lett.* 30, 367-381. doi: [10.1080/00032719708002809](https://www.x-mol.com/paperRedirect/1416526988955033600).

Rameshkumar, P., Ramaraj, R. (2014). Electroanalysis of nitrobenzene derivatives and nitrite ions using silver nanoparticles deposited silica spheres modified electrode. *J. Electroanal. Chem.* 731, 72-77. doi: [10.1016/j.jelechem.2014.08.010](https://www.x-mol.com/paperRedirect/1233825157855858688).

Rastogi, P. K., Ganesan, V., Krishnamoorthi, S. (2014). Palladium nanoparticles incorporated polymer-silica nanocomposite based electrochemical sensing platform for nitrobenzene detection. *Electrochim. Acta* 147, 442-450. doi: [10.1016/j.electacta.2014.09.128](https://www.x-mol.com/paperRedirect/3986982).

Ruiz-Ramirez, M. M., Silva-Carrillo, C., Hinostroza-Mojarro, J. J., Rivera-Lugo, Y. Y., Valle-Trujillo, P., Trujillo-Navarrete, B. (2021). Electrochemical sensor for determination of nitrobenzene in aqueous solution based on nanostructures of TiO_2_/GO. *Fuel* 283, 119326. doi: [10.1016/j.fuel.2020.119326](https://www.x-mol.com/paperRedirect/1534052961153257472).

Sangili, A., Annalakshami, M., Chen, S. M., Chen, T. W., Kumaravel, S., Govindasami, M. (2018). A Facile synthesis of ultra-small cerium oxide nanoparticles for enhanced electrochemical detection of nitrobenzene in water samples. *Int. J. Electrochem. Sci.* 13, 6135-6143. doi: [10.20964/2018.06.118](https://www.x-mol.com/paperRedirect/1372010017246887936).

Shivakumar, M., Dharmaprakash, M. S., Manjappa, S., Nagashree, K. L. (2020). Green synthesis of silver nanoparticles (SNPs)-modified electrode for electrochemical detection of nitrobenzene. *J. Iran. Chem. Soc.* 17, 893-900. doi: [10.1007/s13738-019-01822-z](https://www.x-mol.com/paperRedirect/1225007384705417216).

Singh, S., Devi, P., Singh, D., Jain, D.V., Singla, M. L. (2012). Sensing behavior of silica-coated Au nanoparticles towards nitrobenzene. *Gold Bull.* 45, 75-81. doi: [10.1007/s13404-012-0050-0](https://www.x-mol.com/paperRedirect/1225017936152412163).

Sukanya, R., Sakthivel, M., Chen, S. M., Chen, T. W. (2018). A new type of terbium diselenide nano octagon integrated oxidized carbon nanofiber: An efficient electrode material for electrochemical detection of morin in the food sample. *Sensor. Actuat. B Chem.* 269, 354-367. doi: [10.1016/j.snb.2018.05.013](https://www.x-mol.com/paperRedirect/659003)

Velmurugan, M., Karikalan, N., Chen, S. M., Dai, Z. C. (2017). Studies on the influence of β-cyclodextrin on graphene oxide and its synergistic activity to the electrochemical detection of nitrobenzene. *J. Colloid Interf. Sci.* 490, 365-371. doi: [10.1016/j.jcis.2016.11.036](https://www.x-mol.com/paperRedirect/5340941).

Vinoth, S., Rajaitha, P. M., Pandikumar, A. (2020). In-situ pyrolytic processed zinc stannate incorporated graphitic carbon nitride nanocomposite for selective and sensitive electrochemical determination of nitrobenzene. *Compos. Sci. Technol.* 195, 108192. doi: [10.1016/j.compscitech.2020.108192](https://www.x-mol.com/paperRedirect/1252055050194149376).

Wu, H., Fan, Suhua., Jin, X., Zhang, H., Chen, H., Dai, Z., Zou, X. (2014). Construction of a Zinc Porphyrin−fullerene Derivative Based Non-enzymatic Electrochemical Sensor for Sensitive Sensing of Hydrogen Peroxide and Nitrite. *Anal. Chem.* 86, 6285–6290. doi: 10.1021/ac500245k.

Wu, H., Wei, T., Li, X., Yang, J., Zhang, J., Fan, S., Zhang, H. (2017). Synergistic-effect-controlled tetraoctylammonium bromide/multi-walled carbon nanotube@hemin hybrid material for construction of electrochemical sensor. *J. Electrochem. Soc.* 164, B147−B151. doi: 10.1149/2.1431704jes.

Xu, G., Li, B., Wang, X., Luo, X. (2014). Electrochemical sensor for nitrobenzene based on carbon paste electrode modified with a poly (3, 4-ethylenedioxythiophene) and carbon nanotube nanocomposite. *Microchim. Acta* 181, 463-469. doi: [10.1007/s00604-013-1136-y](https://www.x-mol.com/paperRedirect/1335459675499884573).

Yadav, D.K., Ganesan, V., Sonkar, P. K., Gupta, R., Rastogi, P. K. (2016). Electrochemical investigation of gold nanoparticles incorporated zinc-based metal-organic framework for selective recognition of nitrite and nitrobenzene. *Electrochim. Acta* 200, 276-282. doi: [10.1016/j.electacta.2016.03.092](https://www.x-mol.com/paperRedirect/1233679763637293056).

Yuan, C., Li, N., Zhang, X., Wang, Y., Zhou, S., Zhang, L., et al. (2023). Flower-like copper sulfide-decorated boron-nitrogen co-doped carbon-modified glassy carbon electrode for selective and sensitive electrochemical detection of nitrobenzene in natural water. *Colloid. Surface. A* 675, 132011. doi: [10.1016/j.colsurfa.2023.132011](https://www.x-mol.com/paperRedirect/1677836808768700416).

Zhang, H. K., Liang, S. X., Liu. S. J. (2007). Determination of nitrobenzene by differential pulse voltammetry and its application in wastewater analysis. *Anal. Bioanal. Chem.* 387, 1511-1516. doi:  [10.1007/s00216-006-1014-0](https://www.x-mol.com/paperRedirect/3477955).

Zhang, Y., Zeng, L., Bo, X., Wang, H., Guo, L. (2012). Electrochemical study of nitrobenzene reduction using novel Pt nanoparticles/macroporous carbon hybrid nanocomposites. *Anal. Chim. Acta* 752, 45-52. doi: [10.1016/j.aca.2012.09.040](https://www.x-mol.com/paperRedirect/3588648).

Zhou, Z. Y., Mukherjee, S., Hou, S. J., Li, W. J., Elsner, M., Fischer, R. (2021). Porphyrinic MOF film for multifaceted electrochemical sensing. *Angew. Chem. Int. Ed.* 60, 20551−20557. doi: [10.1002/anie.202107860](https://www.x-mol.com/paperRedirect/1415439140352364544).
